# Crystallography, Molecular Modeling, and COX-2 Inhibition Studies on Indolizine Derivatives

**DOI:** 10.3390/molecules26123550

**Published:** 2021-06-10

**Authors:** Katharigatta N. Venugopala, Sandeep Chandrashekharappa, Christophe Tratrat, Pran Kishore Deb, Rahul D. Nagdeve, Susanta K. Nayak, Mohamed A. Morsy, Pobitra Borah, Fawzi M. Mahomoodally, Raghu Prasad Mailavaram, Mahesh Attimarad, Bandar E. Aldhubiab, Nagaraja Sreeharsha, Anroop B. Nair, Osama I. Alwassil, Michelyne Haroun, Viresh Mohanlall, Pottathil Shinu, Rashmi Venugopala, Mahmoud Kandeel, Belakatte P. Nandeshwarappa, Yasmine F. Ibrahim

**Affiliations:** 1 Department of Pharmaceutical Sciences, College of Clinical Pharmacy, King Faisal University, Al-Ahsa 31982, Saudi Arabia; ctratrat@kfu.edu.sa (C.T.); momorsy@kfu.edu.sa (M.A.M.); mattimarad@kfu.edu.sa (M.A.); baldhubiab@kfu.edu.sa (B.E.A.); sharsha@kfu.edu.sa (N.S.); anair@kfu.edu.sa (A.B.N.); mharoun@kfu.edu.sa (M.H.); 2 Department of Biotechnology and Food Technology, Durban University of Technology, Durban 4001, South Africa; vireshm@dut.ac.za; 3 Department of Medicinal Chemistry, National Institute of Pharmaceutical Education and Research (NIPER-R) Raebareli, Lucknow UP 226002, India; 4 Institute for Stem Cell Science and Regenerative Medicine, NCBS, TIFR, GKVK, Bellary Road, Bangalore 560065, India; 5 Faculty of Pharmacy, Philadelphia University, Amman 19392, Jordan; prankishore1@gmail.com; 6 Department of Chemistry, Visvesvaraya National Institute of Technology, Nagpur 440010, Maharashtra, India; rahulnagdeve3@gmail.com (R.D.N.); sknayak@chm.vnit.ac.in (S.K.N.); 7 Department of Pharmacology, Faculty of Medicine, Minia University, El-Minia 61511, Egypt; yasmine.ibrahim@mu.edu.eg; 8 Pratiksha Institute of Pharmaceutical Sciences, Chandrapur Road, Panikhaiti, Guwahati 781026, Assam, India; pobitrab.phe15@itbhu.ac.in; 9 Department of Health Sciences, Faculty of Medicine and Health Sciences, University of Mauritius, Réduit 80835, Mauritius; f.mahomoodally@uom.ac.mu; 10 Department of Pharmaceutical Chemistry, Shri Vishnu College of Pharmacy, Vishnupur, Bhimavaram 534202, India; raghumrp@svcp.edu.in; 11 Department of Pharmaceutics, Vidya Siri College of Pharmacy, Off Sarjapura Road, Bangalore 560035, India; 12 Department of Pharmaceutical Sciences, College of Pharmacy, King Saud bin Abdulaziz University for Health Sciences, Riyadh 11481, Saudi Arabia; wassilo@ksau-hs.edu.sa; 13 Department of Biomedical Sciences, College of Clinical Pharmacy, King Faisal University, Al-Ahsa 31982, Saudi Arabia; spottathail@kfu.edu.sa; 14 Department of Public Health Medicine, Howard College Campus, University of KwaZulu-Natal, Durban 4001, South Africa; rashmivenugopala@gmail.com; 15 Department of Biomedical Sciences, College of Veterinary Medicine, King Faisal University, Al-Ahsa 31982, Saudi Arabia; mkandeel@kfu.edu.sa; 16 Department of Pharmacology, Faculty of Veterinary Medicine, Kafrelsheikh University, Kafrelsheikh 33516, Egypt; 17 Department of Studies in Chemistry, Shivagangotri, Davangere University, Davangere, Karnataka 577007, India; belakatte@davangereuniversity.ac.in

**Keywords:** indolizine derivatives, molecular modeling, COX-2 inhibition, crystal structure, Hirshfeld surface analysis, energy framework

## Abstract

The cyclooxygenase-2 (COX-2) enzyme is an important target for drug discovery and development of novel anti-inflammatory agents. Selective COX-2 inhibitors have the advantage of reduced side-effects, which result from COX-1 inhibition that is usually observed with nonselective COX inhibitors. In this study, the design and synthesis of a new series of 7-methoxy indolizines as bioisostere indomethacin analogues (**5a**–**e**) were carried out and evaluated for COX-2 enzyme inhibition. All the compounds showed activity in micromolar ranges, and the compound diethyl 3-(4-cyanobenzoyl)-7-methoxyindolizine-1,2-dicarboxylate (**5a**) emerged as a promising COX-2 inhibitor with an IC_50_ of 5.84 µM, as compared to indomethacin (IC_50_ = 6.84 µM). The molecular modeling study of indolizines indicated that hydrophobic interactions were the major contribution to COX-2 inhibition. The title compound diethyl 3-(4-bromobenzoyl)-7-methoxyindolizine-1,2-dicarboxylate (**5c**) was subjected for single-crystal X-ray studies, Hirshfeld surface analysis, and energy framework calculations. The X-ray diffraction analysis showed that the molecule (**5c**) crystallizes in the monoclinic crystal system with space group *P* 2_1_/n with *a* = 12.0497(6)Å, *b* = 17.8324(10)Å, *c* = 19.6052(11)Å, *α* = 90.000°, *β* = 100.372(1)°, *γ* = 90.000°, and V = 4143.8(4)Å^3^. In addition, with the help of *Crystal Explorer* software program using the B3LYP/6-31G(d, p) basis set, the theoretical calculation of the interaction and graphical representation of energy value was measured in the form of the energy framework in terms of coulombic, dispersion, and total energy.

## 1. Introduction

Nonsteroidal anti-inflammatory drugs (NSAIDs) are one of the most commonly prescribed drugs for the treatment of inflammatory conditions worldwide [1]. NSAIDs exhibit their anti-inflammatory activity via inhibition of cyclooxygenase (COX), an enzyme involved in the biosynthesis of prostaglandin G2 (PGG2). By inhibiting the formation of PGG2, the pathway, which would ultimately lead to the inflammatory response, is blocked [2,3]. The COX enzyme has two important isoforms, namely, COX-1 and COX-2. The COX-1 isoform represents the constitutive type that is normally expressed in various regions of the body, such as the kidney and the gastrointestinal tract (GIT), where it is responsible for maintaining certain physiological functions including protection of the gastric mucosa [4,5,6,7]. On the other hand, the COX-2 isoform represents the inducible type that is expressed in response to various inflammatory stimuli and certain substances such as mitogens and cytokines that are produced during injuries. Nonselective NSAIDs that inhibit both COX-1 and COX-2 can have severe undesirable side-effects due to inhibition of the customarily expressed isoform, COX-1. For example, GIT ulceration is a commonly observed adverse effect associated with the use of nonselective NSAIDs [8,9]. Thus, in order to develop anti-inflammatory agents with reduced side-effects, compounds with high selectivity for inhibiting the COX-2 isoform over the COX-1 isoform are required.

Synthetic indolizine derivatives have been shown to interact with a wide range of drug targets such as calcium channels [10], histamine receptors [11], and phospholipase A2 [12]. In addition, they have shown numerous pharmacological properties [13] such as analgesic [14], COX-2-inhibitory [15,16,17,18], anticancer [19,20], antidiabetic [21], antihistaminic [11], antileishmanic [22], antimicrobial [23], antimutagenic [24], antioxidant [25], antiviral [26], larvicidal [27,28], herbicidal [29], antitubercular [30,31,32,33,34,35,36,37], and alpha-7 nicotinic acetylcholine receptor (α-7 nAChR)- [38], *N*-meningitidis-, *N*-acetylneuraminic acid synthese (NmeNANAS)-inhibitory activities [39].

**Figure 1:**
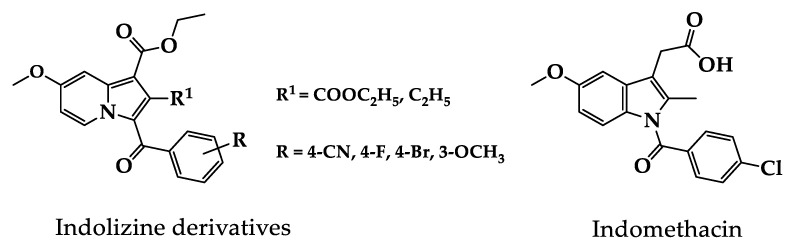
The proposed 7-methoxy indolizine analogues and commercially available nonselective cyclooxygenase (COX) inhibitor (indomethacin) for COX-2 inhibition action.

**Scheme 1:**
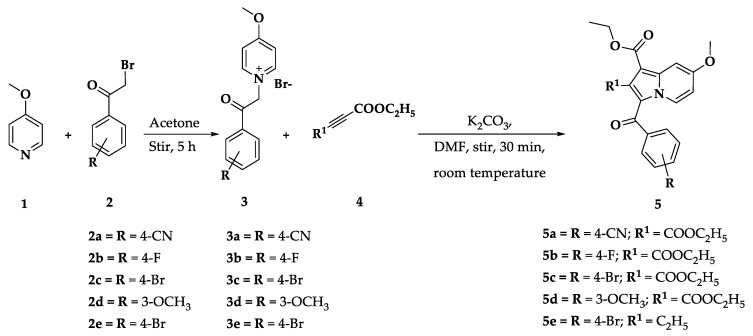
Synthetic outline for the construction of diethyl 7-methoxy-3-(3-substituedbenzoyl)indolizine-1,2-dicarboxylates (**5a**–**d**) [35] and ethyl 3-(4-bromobenzoyl)-2-ethyl-7-methoxyindolizine-1-carboxylates (**5e**).

In persistence of our interest in pharmacologically active heterocyclic compounds [40,41,42,43,44,45,46,47,48], polymorphism studies [49,50,51,52], and the discovery of anti-inflammatory agents [53,54,55,56,57,58,59], in the present investigation, we synthesized a range of 7-methoxy indolizine derivatives as indomethacin analogues (Figure 1 and Scheme 1) to evaluate their potential as anti-inflammatory agents. The title compounds (**5a**–**e**) were evaluated for their pharmacological activity against the COX-2 enzyme in order to study the influence of ethyl ester at the 1- and 2-positions, ethyl at the 2-position, and the diverse substituent on the benzoyl ring at the 3-positions of the indolizine core on the biological action.

## 2. Results and Discussion

### 2.1. Chemistry

**Figure 2:**
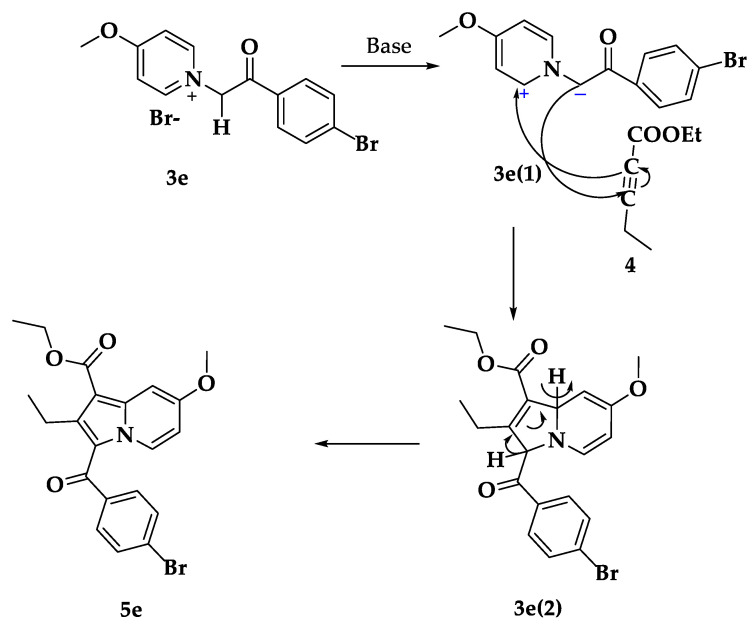
Plausible reaction mechanism for the construction of ethyl 3-(4-bromobenzoyl)-2-ethyl-7-methoxyindolizine-1-carboxylate (**5e**) [35,60].

The design of the target compounds (**5a**–**e**) was mainly based on the close chemical structural relationship with the commercially available NSAIDs indomethacin (Figure 1). The synthesis of the target compounds is depicted in Scheme 1. The intermediates (**3a**–**e**) were obtained by stirring a mixture of 4-methoxy pyridine and *para*- and *meta*-substituted phenacyl bromides in acetone medium at 5 h and were then further reacted with diethyl but-2-ynedioate in the existence of potassium carbonate in dimethylformamide solvent medium for 30 min. The resulting title compounds were purified by column chromatography using mixture of ethyl acetate and hexane as an eluent, and the purity of the compounds was more than 99% with a satisfactory yield (69% to 77%). The physicochemical property of the target compound ethyl 3-(4-bromobenzoyl)-2-ethyl-7-methoxyindolizine-1-carboxylate (**5e**) is presented in Table 1. The chemical structure of this compound (**5e**) was confirmed with spectroscopic techniques such as FT-IR, ^1^H-NMR and ^13^C-NMR, and LC–MS. The Fourier-transform infrared (FT-IR) spectroscopy revealed benzoyl and ester carbonyl groups at 1699 and 1668 cm^−1^, respectively (spectra are available as Electronic Supplementary Materials). The ^1^H-NMR spectra revealed the methoxy group at 3.86 ppm, ester peak triplet appearance of 1.34 ppm, and ester group quartet appearance of 4.30 ppm. The ^13^C-NMR spectra revealed the appearance of benzoyl and ester carbonyl groups at 186.00 and 166.07 ppm, respectively. The molecular ion peaks of this compound (**5e**) were in good agreement with its molecular mass. Title compounds diethyl 3-(4-cyanobenzoyl)-7-methoxyindolizine-1,2-dicarboxylate (**5a**), diethyl 3-(4-fluorobenzoyl)-7-methoxyindolizine-1,2-dicarboxylate (**5b**), diethyl 3-(4-bromobenzoyl)-7-methoxyindolizine-1,2-dicarboxylate (**5c**), and diethyl 7-methoxy-3-(3-methoxybenzoyl)indolizine-1,2-dicarboxylate (**5d**) were resynthesized [35], and their physicochemical properties are presented in Table 1. The proposed general reaction mechanism for the target compound (**5e**) is illustrated in Figure 2.

The construction of the final indolizine compound (**5e**) was achieved via 1,3-dipolar cycloaddition of intermediate pyridinium salt **3e**, generating ylide **3e**(**1**) using a base. This ylide carbanion subsequently attacks the electron-deficient acetylene triple bond of reactant (**4**). This triple bond anion then attacks the carbocation of compound **3e**(**1**), yielding compound **3e**(**2**). This loss of hydrogen through oxidation leads to the construction of indolizine nucleus **5e** (Figure 2).

### 2.2. Crystallography

**Figure 3:**
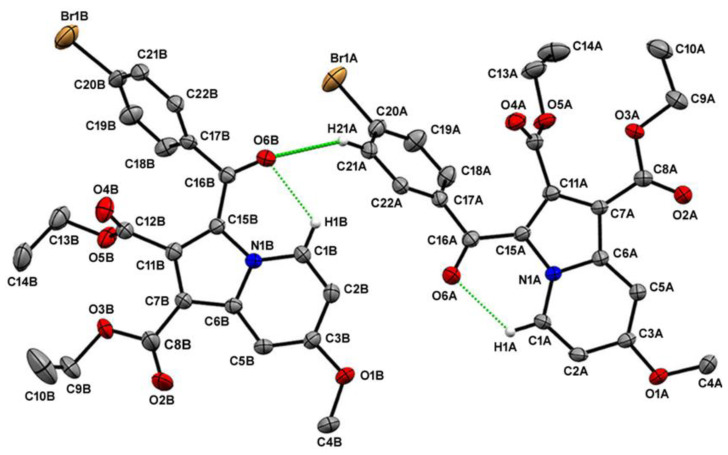
The ORTEP of **5c** at 50% ellipsoidal probability and atom labeling of non-hydrogen atoms. The intramolecular C–H···O interactions are shown as green dotted lines.

**Figure 4:**
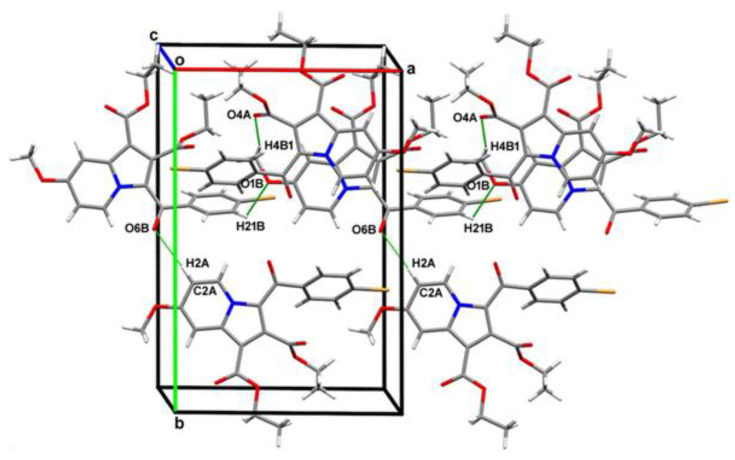
Crystal packing of **5c** prefers the weak C–H···O interactions.

**Figure 5:**
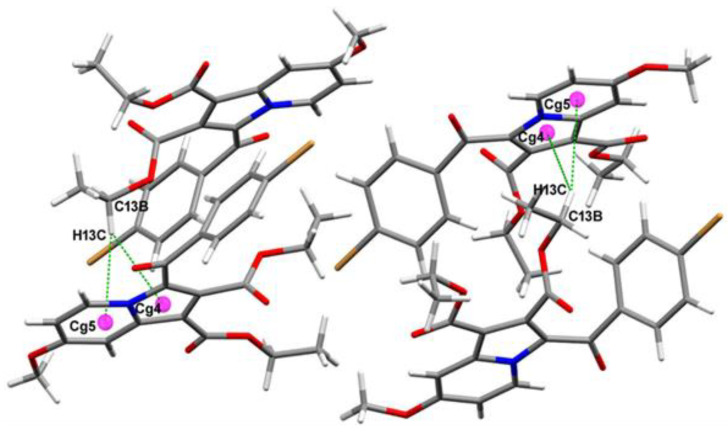
The C–H···π contacts exist in **5c** crystal structure in its molecular assembly.

The crystallographic details of **5c** are listed in Table 2. The crystal structure of **5c** crystallized in the monoclinic space group *P* 2_1_/n with eight molecules in the unit cell. The lattice parameters were *a* = 12.0497(6)Å, *b* = 17.8324(10)Å, *c* = 19.6052(11)Å, *α* = 90.000°, *β* = 100.372(1)°, *γ* = 90.000°, and V = 4143.8(4)Å^3^. The asymmetric unit of **5c** crystal structure showed two molecules which preferred the intramolecular C–H···O interactions shown as a dotted line and 50% thermal ellipsoidal probability of non-hydrogen atoms (Table 3 and Figure 3). There were eight molecules in the unit cell of 5c, where intermolecular weak hydrogen bonds C2A–H2A···O6B and C4B–H4B1···O4A were the major interactions, along with C–H···π interactions that stabilized the molecular assembly, as shown in Figure 4 and Figure 5 (Table 3).

### 2.3. Hirshfeld Surface Analysis

**Figure 6:**
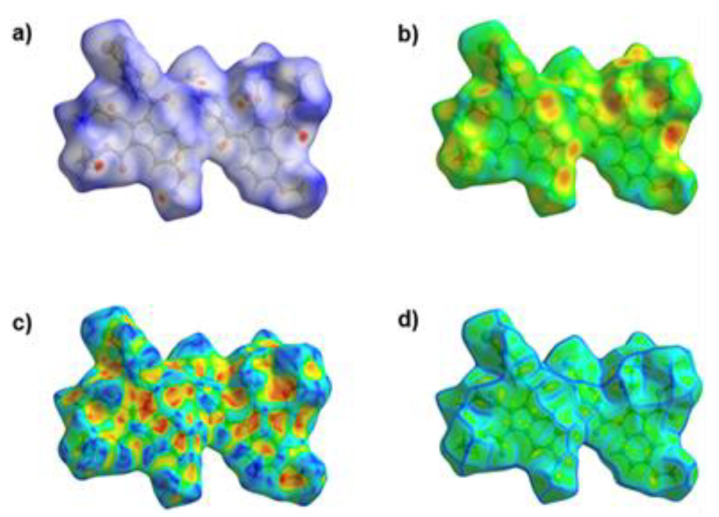
Hirshfeld surfaces of **5c** mapped with (**a**) d_norm_ (**b**) d_e_ (**c**) shape index, and (**d**) curvedness.

**Figure 7:**
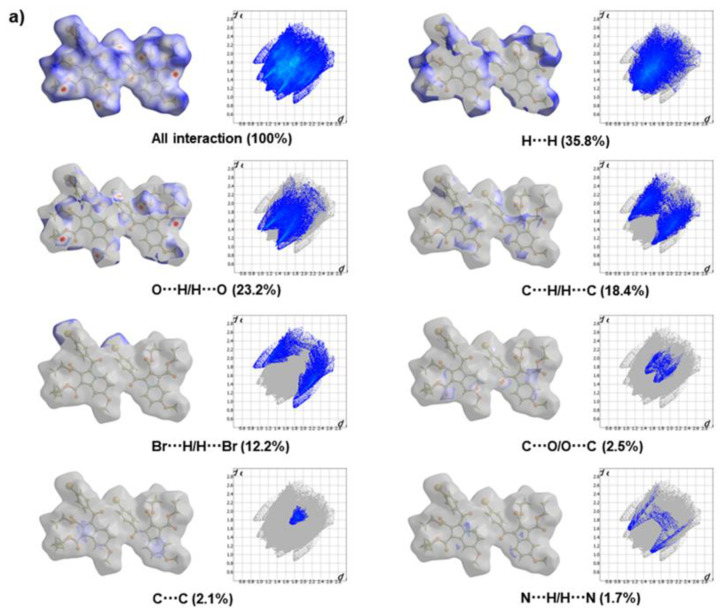

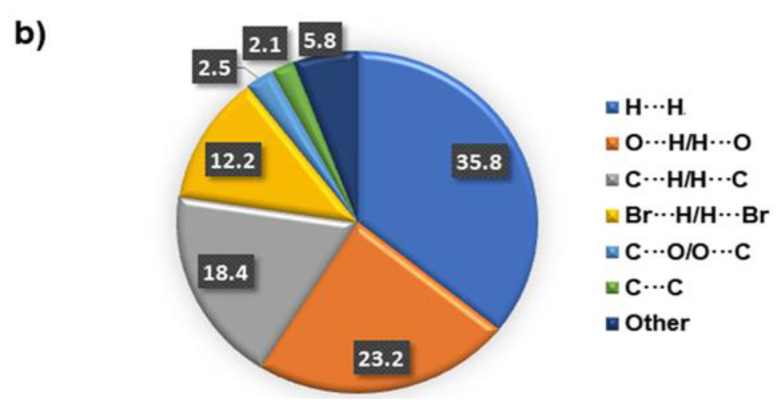
(**a**) The 2D fingerprint plots of the compound **5c** with a percentage of interaction. (**b**) The short contact contributions derived from H···H, O···H/H···O, C···H/H···C, Br···H/H···Br, O···C/C···O, and C···C contacts. The values mentioned in the pie chart are in percentage form.

The Hirshfeld surfaces of the crystal structure **5c** were investigated to illustrate the nature of intermolecular interactions and visualization of intermolecular close contacts in its crystal structure using *Crystal Explorer 17.5* [60], which mapped over d_e_, d_norm_, shape index, and curvedness, as shown in Figure 6. The contribution of individual intermolecular interactions on the Hirshfeld surface can be defined by color codes. On the d_norm_ surface, the red color shows the shorter molecular contacts and the blue color on the d_norm_ surface area represents the longer molecular contacts. The white color on the d_norm_ surface indicates the contact around the van der Waals radii. In the d_norm_ surfaces, the red color shows the hydrogen bonding H···O contacts, whereas the blue surface area represents the H···H contacts (Figure 6a). The d_e_ surface features appear as a relatively flat green region where the contact distances are similar (Figure 6b). The adjacent highlighted red and yellow regions on the shape index surface also show the strong hydrogen bonding interactions present in the molecule (Figure 6c), whereas the blue curved and yellow regions on the curvedness surfaces shows the H···H interactions (Figure 6d). The 2D fingerprint plots [61] show the sharp spike, which represents the intermolecular interactions present in the molecule (Figure 7a). Again, it shows that C–H···O interactions were predominant (23.2%) after the H···H contacts, which led to the highest contribution of 35.8% in comparison to other interactions, suggesting that weak C–H···O hydrogen bonding plays an essential role in its crystal packing. The percentage contributions of other intermolecular interactions in this crystal structure were as follows: C···H/H···C (18.4%), H···Br/Br···H (12.2%), C···O/O···C (2.5%), C···C (2.1%), N···H/H···N (1.7%), etc. (Figure 7b).

### 2.4. Energy Framework Calculation

**Figure 8:**
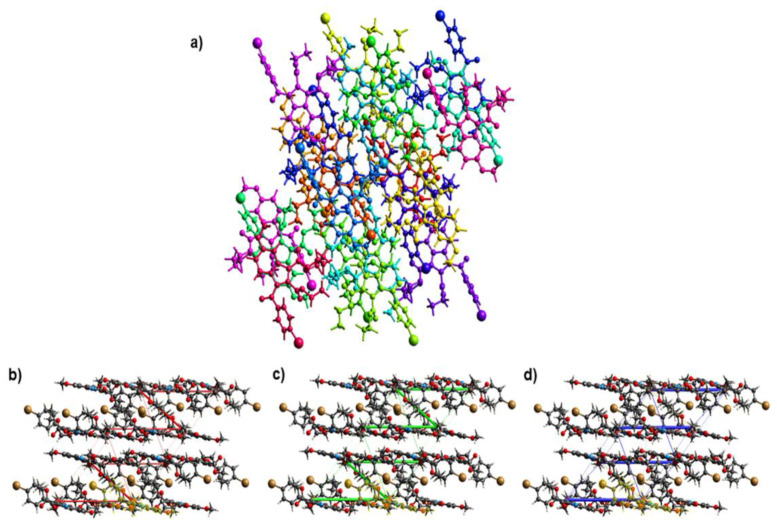
(**a**) Selected molecules for **5c** present within 3.8 Å and cylindrical tube formation for the coulombic energy as red tubes (**b**), for dispersion energy as green tubes (**c**), and total energy as blue tubes (**d**).

Furthermore, the *Crystal Explorer 17.5* software was used to evaluate the interaction energies for the crystal structure **5c**. Energy frameworks have a strong and remarkable way of imagining the supramolecular existence of molecular crystal structures. The interaction energies between the molecules are obtained using monomer wave functions at the B3LYP/6-31G (d, p) level. [62]. As prescribed, the tube size used in all the energy frameworks was 80 (scale factor), and the cutoff for the energy threshold value was set to zero. In the 3D topological images, the diameter of the tube cylinder reflects the interaction energy in the molecular packing for the corresponding interaction. The molecules present within the 3.8 Å circle within 1 × 1 × 1 unit cell dimensions were selected for this calculation (Figure 8a).

Energies between molecular pairs are expressed as cylinders that connect molecular pair centroids with a cylindrical radius proportional to the energy interaction magnitude. The energy framework was outlined as red cylinders for E_elec_, green cylinders for E_dis_, and blue cylinders for E_tot_, as shown in (Figure 8b–d), and the relative strength of molecular packing was expressed in various directions by these tubes. The supramolecular nature of the crystal structure was, thus, visualized by energy structures in a special way. The calculated energy values are listed in Table 4 for electrostatic, polarization, dispersion, and total interaction energy, which suggests that **5c** crystal structure preferred dispersion energy over others.

### 2.5. Pharmacology

The COX-2-inhibitory activity of the target compounds (**5a**–**5e**) is presented in Table 5. As can be seen from Table 5, all the indolizines displayed interesting inhibitory activity against COX-2 similar to the commercially available drug indomethacin. Compound **5a** with a 4-cyanobenzoyl group attached to the 3-position of the indolizine scaffold, having two ethyl carboxylate groups attached to the 1- and 2-position of the scaffold, emerged as the most promising compound with the highest COX-2-inhibitory activity (IC_50_ = 5.84 µM). Replacement of the electron-withdrawing nitrile group with halogens such as fluorine and bromine atoms at the 4-position of the benzoyl ring exhibited detrimental COX-2 inhibitory activity for compounds **5b** and **5c** with IC_50_ values 6.73 µM and 6.99 µM, respectively. It is remarkable to note that title compound **5e** with only one ethyl ester moiety at the first position of the indolizine pharmacophore exhibited a further reduction in activity (IC_50_ = 7.38 µM) as compared to the structurally similar compound **5c** (IC_50_ = 6.99 µM).

The compound **5d**, substituted with a methoxy functional group at the 3-position of the benzoyl ring, displayed the least inhibitory activity (IC_50_ = 8.49 µM). In general, the availability of the electron-withdrawing functional groups at the *para* position of the benzoyl ring was found to be favorable for COX-2-inhibitory activity as compared to the presence of the electron-donating functional groups at the *meta* position. Previously these derivatives demonstrated excellent safety profiles [35]; thus, they could be considered as lead molecules for further improvement of novel potential COX-2 inhibitors.

### 2.6. Computational Studies

Molecular docking studies are considered an invaluable in silico approach to correlate the in vitro structure–activity relationship (SAR) of chemical compounds [63]. To gain insight into the inhibitory activity of indolizines (**5a**–**e**), we investigated their key interactions with the COX-2 receptor through a computational approach. The docking study was conducted with Accelrys Discovery Studio Client 4.0 software. The docking interaction energies and the residue interactions of indolizines **5a**–**e** and indomethacin are reported in Table 6. All the compounds demonstrated favorable docking energy ranging from −38.22 to −53.29 kcal/mol, indicating that they have a good binding affinity with the COX-2 receptor, as demonstrated from their biological activities.

**Figure 9:**
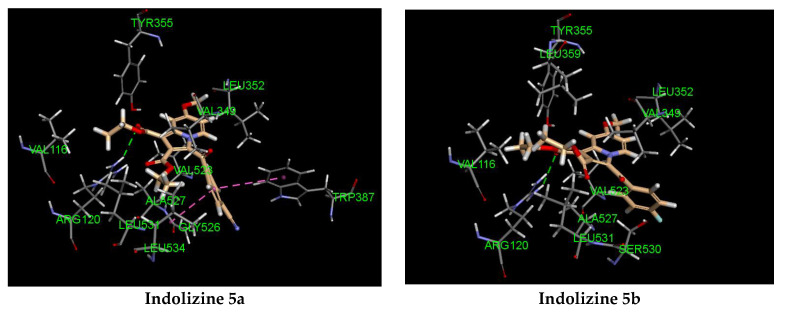

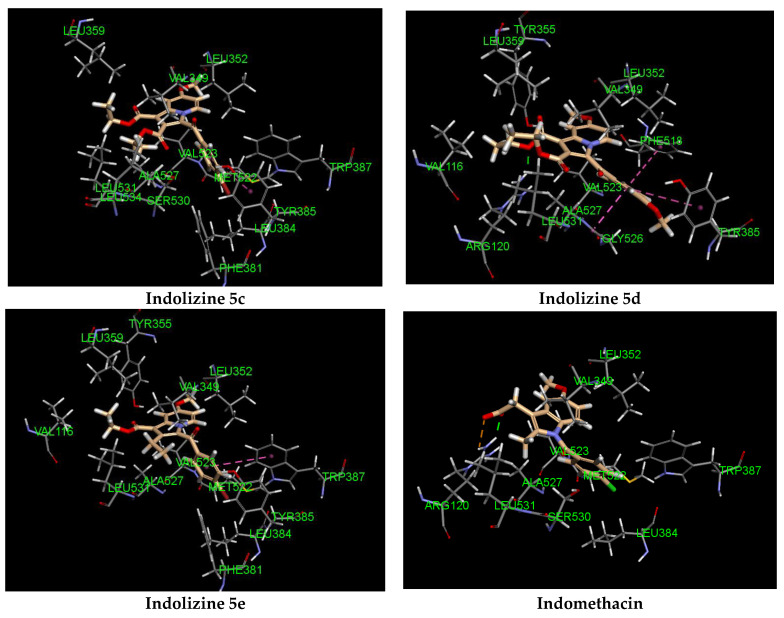
Predicted docking pose of indolizines (**5a**–**e**) and indomethacin (salmon-filled spheres) in the cyclooxygense-2 COX-2 domain (PDB 4COX). Hydrogen bonding and pi–pi interactions are represented in green and violet dotted lines, respectively.

The predicted docking poses of indolizines **5a**–**e** and indomethacin are depicted in Figure 9. All the compounds adopted a similar conformation to that of indomethacin, where the indolizine ring is taken into a sandwich between the amino-acid residues Ala527, Val523 and Val349, Leu352. The benzoyl ring is oriented toward the residues Tyr385 and Trp387, while the methoxy group is located in the deep region of the receptor. As can be observed from the binding poses, indolizines **5a**, **5b**, and **5d** demonstrated favorable hydrogen bonding interaction between Arg120 with the ester group at the 1-position of the indolizine scaffold, while indomethacin showed hydrogen bonding and ionic bonding interactions between the same residue Arg120 and its carboxylic acid group. This indicated that the ionic interaction with Arg120 is not a requirement for maintaining the potency of the compounds. Furthermore, the indolizines **5c** and **5e** containing a bromine atom at the *para* position of the benzoyl ring showed no hydrogen bonding involvement with the residue Arg120. Therefore, the major contribution to the COX-2 activity of our compounds principally involves hydrophobic interactions with the indolizine ring and with the substituents at positions 2 and 3 of indolizine. It can be noted that only the bromine substituent on the benzoyl ring (**5c** and **5e**) demonstrated hydrophobic interactions with residues Leu384 and Met522, while pi–pi interactions were observed for all indolizines with the exception of fluoro indolizine **5b** and indomethacin. Therefore, the substituent in the benzoyl ring has a minor contribution to the activity of indolizine. However, it has been demonstrated that the benzoyl ring on indomethacin is important to bioactivity since the replacement of *N*-benzoyl by *N*-benzyl led to a reduction in COX-2 inhibition [64].

The molecular modeling study provided insight into the structural requirement of indolizines for COX-2-inhibitory activity. Moreover, indolizines **5a**–**e** are more likely to be selective COX-2 inhibitors. It has been validated that COX-1 and COX-2 selectivity is mainly due to the ionic interaction with residue Arg120 since the corresponding ester and amide of indomethacin derivatives presented good selectivity in favor of COX-2 inhibition [64].

## 3. Materials and Methods

### 3.1. Chemistry

All the commercially offered chemicals and solvents were purchased from Sigma-Aldrich Co. (St. Louis, MO, USA). All the chemical reactions were performed in hot-air-dried glassware in the presence of a nitrogen atmosphere consuming dry solvents. A Shimadzu FT-IR spectrophotometer (Columbia, MD, USA) was used to record the FT-IR spectra. Furthermore, ^1^H- and ^13^C-NMR spectra were documented at ambient temperature on Bruker AVANCE III 400 MHz instruments (San Jose, CA, USA) using CDCl_3_ and DMSO-*d*_6_ as solvents. An Agilent 1200 series instrument (Santa Clara, CA, USA) in conjunction with a 6140 single-quadrupole mass spectrometer using positive and negative ESI mode with a mass selective detector (MSD) range of 100–2000, as well as 0.1% aqueous trifluoroacetic acid in an acetonitrile system on the C18-BDS column, was used to record liquid chromatography–mass spectrometry (LC–MS) spectra. Then, an elemental analysis was carried out using the analyzer FLASH EA 1112 CHN (Thermo Finnigan LLC, New York, NY, USA). A single-crystal X-ray diffraction study was performed using a Bruker KAPPA APEX II DUO diffractometer (Madison, WI, USA) equipped with a charge-coupled device (CCD) detector; monochromated Mo Kα radiation (λ = 0.71073 Å) was used. Data collection was carried out using an Oxford Cryostream cooling system featuring the Bruker Apex II software (Madison, WI, USA) at 173(2) K [16].

### 3.2. General Synthetic Procedure for 4-methoxy-1-(2-(substituted phenyl)-2-oxoethyl)pyridinium Bromides (**3a**–**e**)

To a solution of 4-methoxypyridine (**1**) (0.0091 mol, 1 g) in dry acetone solvent (10 mL), substituted-phenacylbromide (0.0091 mol, 2.03 g) was added and agitated at room temperature for 5 h. The completion of the reaction was observed on thin-layer chromatography (TLC). The product obtained was separated, filtered, and desiccated under vacuum to yield 92–99% 1-(2-(substituted phenyl)2-oxoethyl)-4-methoxypyridinium bromides. 

### 3.3. Synthetic Procedure for the Synthesis of Ethyl 3-(4-bromobenzoyl)-2-ethyl-7-methoxyindolizine-1-carboxylate (**5e**)

To a stirred solution of 1-(2-(4-bromophenyl)-2-oxoethyl)-4-methoxypyridinium bromide (**3e**) (0.0026 mol, 1 g), in dry dimethylformamide, ethyl pent-2-ynoate (**4**) (0.0025 mol, 0.512 g) and K_2_CO_3_ (0.0051 mol, 0.713 g) were added. It was stirred at room temperature for 30 min. The completion of the reaction was monitored on TLC. After completion of the reaction, the solvent was evaporated under reduced pressure and diluted with ethyl acetate. The organic layer was washed with water, brine, and dried with sodium sulfate. The crude compound was purified by column chromatography to afford a 69% yield of compound **5e**. The physicochemical characteristics are tabulated in Table 1. Appearance: light-yellow crystalline compound. FT-IR (KBR neat cm^−1^): 1699, 1668, 1639, 1602. ^1^H-NMR (400 MHz CDCl_3_) δ = 9.18 (d, J = 7.2 Hz, 1H), 7.69–7.60 (m, 3H), 7.10–7.05 (m, 2H), 6.57–6.54 (m, 1H), 4.30 (q, J = 7.2 Hz, 2H), 3.86 (s, 3H), 2.57 (q, J = 7.2 Hz, 2H), 1.34 (t, J = 7.2 Hz, 3H), 0.91 (t, J = 7.2 Hz, 3H). ^13^C-NMR (100 MHz CDCl_3_) δ = 186.00, 166.07, 165.01, 163.56, 159.37, 144.79, 142.77, 137.58, 137.55, 130.95, 130.86, 129.61, 121.61, 121.10, 115.64, 115.42, 108.20, 102.82, 97.57, 59.64, 55.57, 20.06, 15.99, 14.41. Analysis calculated for C_21_H_20_BrNO_4_: C, 58.62; H, 4.68; N, 3.26; found: C, 58.69: H, 4.52: N, 3.24. The spectra are available as Electronic Supplementary Materials.

### 3.4. Crystallography

Single-crystal X-ray diffraction data of **5c** were collected on a Bruker KAPPA APEX II DUO diffractometer using graphite-monochromated Mo-Kα radiation (χ = 0.71073 Å). Data collection was carried out at 173(2) K. Oxford Cryostream was used to control temperature (Oxford Cryostat). The cell refinement and data reduction for **5c** were performed using the program SAINT [65], and the absorption correction was performed using SADABS [65].

The crystal structure of **5c** was solved by direct methods using SHELXS-18 [66] and refined by the full-matrix least-squares method based on F^2^ using SHELXL-2018 [66]. The program WinGx [67] was used to prepare molecular graphic images. All non-hydrogen atoms were refined anisotropically, and all hydrogen atoms were placed in idealized positions and refined in riding models with U_iso_ assigned 1.2 or 1.5 times U_eq_ of the parent atoms [67]. The C–H bond distances was constrained to 0.95 Å for aromatic hydrogen and 1.00 Å for methyl hydrogen. The crystallographic parameters are listed in Table 2.

### 3.5. In Vitro COX-2 Inhibition Assay

The title compounds **5a**–**e** were tested for in vitro human recombinant COX-2 enzyme inhibition activity following our previously reported protocol [68].

### 3.6. Computational Studies

The computational study for test compounds **5a**–**e** was conducted with Accelrys Discovery Studio Client 4.0 (Waltham, MA, USA) using the indomethacin crystal structure PDB 4COX following our previously reported protocol [68].

## 4. Conclusions

In this study, a series of diethyl 7-methoxy-3-(3-substituted benzoyl)indolizine-1,2-dicarboxylate derivatives (**5a**–**d**) and ethyl 3-(4-bromobenzoyl)-2-ethyl-7-methoxyindolizine-1-carboxylate (**5e**) were synthesized and evaluated for the inhibition of COX-2 enzyme activity. All the compounds were demonstrated to be active inhibitors of COX-2, with the most active compound (**5a**) having an IC_50_ value comparable to that of indomethacin, a marketed COX inhibitor. Computational studies were conducted to analyze the key interactions of these compounds with the amino-acid residues of the COX-2 receptor. Hydrophobic interactions were observed to be mainly responsible for the inhibitory COX-2 activity of indolizines. The compound **5c** was crystallized in a monoclinic crystal system with space group P 2_1_/n. The molecule was observed to have both intra- and intermolecular hydrogen bonds and exhibited C–H···π interactions for stability. In order to understand and visualize the contribution of different intermolecular interactions, Hirshfeld surface analysis with 2D fingerprint plots was carried out to provide insight into the stability of the crystal structure. In terms of electrostatic, dispersion, and total energy, the systematic and theoretical energy was calculated using the software program *Crystal Explorer*, which further provided 3D topological images. Indolizines **5a**–**e** could be considered as lead compounds for developing novel COX-2 inhibitors.

## Figures and Tables

**Table 1:**
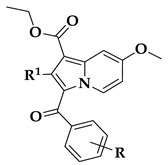
Physicochemical properties of the target compound ethyl 3-(4-bromobenzoyl)-2-ethyl-7-methoxy-indolizine-1-carboxylate (**5e**).

Compound	Mol Formulae (Mol Mass)	R	R^1^	Yield (%) ^a,b^	m.p. (°C)	*c*LogP ^c^
**5a**	C_23_H_20_N_2_O_6_ (420)	4-CN	COOC_2_H_5_	72	171	3.4454
**5b**	C_22_H_20_FNO_6_ (413)	4-F	COOC_2_H_5_	77	147	4.0199
**5c**	C_22_H_20_BrNO_6_ (473)	4-Br	COOC_2_H_5_	73	134	4.7399
**5d**	C_23_H_23_NO_7_ (425)	3-OCH_3_	COOC_2_H_5_	75	142	4.0294
**5e**	C_21_H_20_BrNO_4_ (430)	4-Br	C_2_H_5_	69	108	6.2773

^a^ The target compound was confirmed by physical and spectral data. ^b^ The final yield obtained after purification by column chromatography. ^c^ *c*Log*P* was calculated using ChemBioDraw Ultra 16.0v.

**Table 2:** The crystallographic refinement parameters of **5c** (diethyl 3-(4-bromobenzoyl)-7-methoxyindolizine-1,2-dicarboxylate).

DATA	5c
Formula	C_22_H_20_BrNO_6_
Formula weight	474.30
CCDC	2,045,116
Temperature (K)	173(2)
Wavelength (Å)	0.71073
Crystal system	Monoclinic
Space group	P 2_1_/n
*a* (Å)	12.0479(6)
*b* (Å)	17.8324(10)
*c* (Å)	19.6052(11)
*α* (˚)	90.000
*β* (˚)	100.372(1)
*γ* (˚)	90.000
V (Å^3^)	4143.8(4)
Z’, Z	2, 8
Density (g·cm^−3^)	1.52
μ (mm^−1^)	2.023
F (000)	1936.0
θ (min, max)	1.6, 28.3
h_min_, _max_, k_min_, _max_, l_min_, _max_.	−16, 15; −23, 23; −26, 26
No. of refl.	10,282
No of unique ref./Obs. ref.	10,282/6833
No. parameters	576
R_all_, R_obs_	0.079, 0.041
wR_all_, wR_obs_	0.100, 0.088
Δρ_min_, _max_(eÅ^−3^)	−0.473, 0.393
G.O.O.F.	1.024

**Table 3:** Intra- and intermolecular interactions of **5c**.

D–X···A	D–X (Å)	X···A (Å)	D···A (Å)	<D–X···A (^o^)
C21B–H21B···O1B ^i^	0.95	2.55	3.109(3)	118
C1A–H1A···O6A ^i^	0.95	2.27	2.851(3)	119
C1A–H1A···O6A ^i^	0.95	2.26	2.855(3)	120
C2A–H2A···O6B ^ii^	0.95	2.52	3.466(3)	174
C4B–H4B1···O4A ^iii^	0.98	2.50	3.442(3)	162
C13B–H13C···π ^iv^	0.98(24)	2.92	3.735(3)	140
C13B–H13C···π* ^v^	0.98(24)	2.86	3.785(3)	156

Symmetry codes: (i) 1 + x, y, z; (ii) 3/2 − x, 1/2 + y, 1/2 − z; (iii) −1/2 + x, 1/2 − y, 1/2 + z; (iv) x, y, z; (v) x, y, z; Note: −π and π* are the centroids of the (N1A–C6A–C7A–C11A–C15A) and (N1A–C1A–C2A–C3A–C5A–C6A) aromatic rings, respectively.

**Table 4:** Interaction energies as obtained from the *Crystal Explorer 17.5* (in kJ/mol) for the **5c** compound.

Color	*N*	Symop	R	E_ele	E_pol	E_dis	E_rep	E_tot
	1	-	4.55	−22.8	−5.1	−98.5	37.6	−84.7
	1	-	14.58	−0.5	0	−0.4	0	−0.8
	1	-	11.1	4.2	−5.5	−31.3	16.2	−14.4
	1	-	14.41	0.2	0	−0.3	0	−0.1
	1	-	13.98	0	0	−1.3	0	−1.3
	1	-	17.79	0.2	0	−0.2	0	0
	1	-	11.16	1	−4.4	−22.6	8.9	−15
	1	-	16.73	0.1	0	−0.1	0	0
	1	-	18.83	0.3	0	−0.2	0	0.2
	1	-	12.4	0.8	0	−0.8	0	0.1
	1	−x + 1/2, y + 1/2, −z + 1/2	11.15	−16.2	−1.2	−22.8	49.6	2.5
	1	x + 1/2, −y + 1/2, z + 1/2	12.57	−0.6	0	−0.6	0	−1.2
	1	−x, −y, −z	18.25	0.3	0	−0.1	0	0.2
	2	−x + 1/2, y + 1/2, −z + 1/2	12.35	−0.3	−0.1	−2.1	0	−2.3
	1	x + 1/2, −y + 1/2, z + 1/2	10.75	−2.2	−0.3	−3.1	0	−5.2
	1	x, y, z	12.05	−11.3	−2.6	−26.1	14.3	−25.2
	1	−x, −y, −z	23.43	0.1	0	0	0	0.1
	1	x, y, z	17.83	−0.1	0	−0.2	0	−0.3
	1	−x, −y, −z	16.07	−0.3	0	−0.1	0	−0.4
	1	x + 1/2, −y + 1/2, z + 1/2	22.54	0	0	0	0	0

**Table 5:** In vitro inhibitory study of cyclooxygenase-2 (COX-2) on diethyl 7-methoxy-3-(3-substitued-benzoyl)-indolizine-1,2-dicarboxylates (**5a**–**d**) and ethyl 3-(4-bromobenzoyl)-2-ethyl-7-methoxy-indolizine-1-carboxylate (**5e**).

Compound	Compound Structure	IC_50_ * (µM)
**5a**	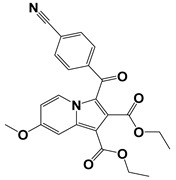	5.84 ± 0.03 *^a^*
**5b**	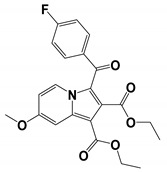	6.73 ± 0.03 *^a,b^*
**5c**	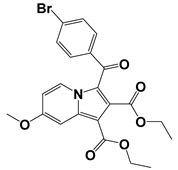	6.99 ± 0.03 *^b^*
**5d**	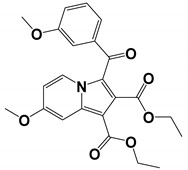	8.49 ± 0.03, *^b,c^*
**5e**	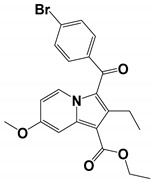	7.38 ± 0.03 *^c,d^*
Indomethacin	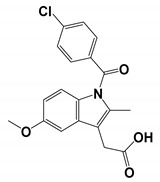	6.84 ± 0.03 *^b,d^*
Celecoxib	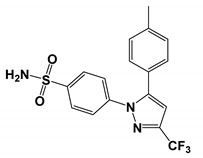	0.05 ± 0.03 *^a^*

* IC_50_ value is defined as the concentration of test and standard substances required to produce 50% inhibition of human recombinant COX-2 enzyme by means of three determinations using the enzyme-linked immune sorbent assay kit. *^a–d^* Title compounds not sharing a letter vary significantly (*p* < 0.05).

**Table 6:**
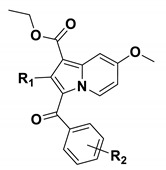
Docking results of indolizines (**5a**–**e**) and indomethacin against cyclooxygense-2 (COX-2) receptor (PDB 4COX).

Entry	R_1_	R_2_	CDocker Interaction Energy	Residues Interaction			
				H-Bonding	pi–pi	pi-alkyl pi-halogen	Alkyl-alkyl Alkyl-halogen
**5a**	CO_2_Et	4-CN	39.51	Arg120	Trp387 Gly526	Val349, Leu352, Ala527, Val523, Tyr355	Val116, Val349, Leu531, Leu534
**5b**	CO_2_Et	4-F	38.22	Arg120		Val349, Leu352, Ala527, Val523, Tyr355	Val116, Leu359, Leu531
**5c**	CO_2_Et	4-Br	46.69	-	Tyr385	Val349, Leu352, Ala527, Val523, Tyr385, Trp387, Phe381	Val116, Val349, Leu359, Leu 531, Leu534, Leu384, Met522
**5d**	CO_2_Et	3-OCH_3_	53.29	Arg120	Tyr385 Trp387 Gly526	Val349, Leu352, Ala527, Val523, Tyr355	Val116, Leu359, Leu531
**5e**	Et	4-Br	48.54	-	Trp387	Val349, Leu352, Ala527, Val523, Tyr385, Trp387, Phe381	Val116, Val349, Leu359, Leu 531, Leu534, Leu384, Met522
Indomethacin			55.36	Arg120 (ionic)		Val349, Leu352, Ala527, Val523, Trp387	Val349, Ala527, Leu531, Leu384, Met522

## Data Availability

Crystal data of compound **5c** were deposited in the Cambridge Crystallographic Data Center (CCDC) (https://www.ccdc.cam.ac.uk/, accessed on 18 September 2020) with number 2045116.

## Supplementary Materials

The following are available online: Figure S1. FT-IR of FT-IR of diethyl 3-(4-cyano benzoyl)7-methoxyindolizine-1,2-dicarboxylate (**5a**); Figure S2. ^1^H-NMR of diethyl 3-(4-cyano benzoyl)7-methoxyindolizine-1,2-dicarboxylate (**5a**); Figure S3. ^13^C-NMR of diethyl 3-(4-cyano benzoyl)7-methoxyindolizine-1,2-dicarboxylate (**5a**); Figure S4. FT-IR of diethyl 3-(4-fluoro benzoyl)7-methoxyindolizine-1,2-dicarboxylate (**5b**); Figure S5. ^1^H-NMR of diethyl 3-(4-fluoro benzoyl)7-methoxyindolizine-1,2-dicarboxylate (**5b**); Figure S6. ^13^C-NMR of diethyl 3-(4-fluoro benzoyl)7-methoxyindolizine-1,2-dicarboxylate (**5b**); Figure S7. FT-IR of diethyl 3-(4-bromobenzoyl)7-methoxyindolizine-1,2-dicarboxylate (**5c**); Figure S8. ^1^H-NMR of diethyl 3-(4-bromobenzoyl)7-methoxyindolizine-1,2-dicarboxylate (**5c**); Figure S9. ^13^C-NMR of diethyl 3-(4-bromobenzoyl)7-methoxyindolizine-1,2-dicarboxylate (**5c**); Figure S10. FT-IR of diethyl 7-methoxy-3-(3-methoxybenzoyl)indolizine-1,2-dicarboxylate (**5d**); Figure S11. ^1^H-NMR of diethyl 7-methoxy-3-(3-methoxy benzoyl)indolizine-1,2-dicarboxylate (**5d**); Figure S12. ^13^C-NMR of diethyl 7-methoxy-3-(3-methoxy benzoyl)indolizine-1,2-dicarboxylate (**5d**); Figure S13. FT-IR of ethyl 3-(4-bromobenzoyl)-2-ethyl-7-methoxyindolizine-1-carboxylate (**5e**); Figure S14. ^1^H-NMR of ethyl 3-(4-bromobenzoyl)-2-ethyl-7-methoxyindolizine-1-carboxylate (**5e**); Figure S15. ^13^C-NMR of ethyl 3-(4-bromobenzoyl)-2-ethyl-7-methoxyindolizine-1-carboxylate (**5e**); Figure S16. checkCIF/PLATON report of checkCIF/PLATON report of diethyl-3-(4-bromo benzoyl)7-methoxyindolizine-1,2-dicarboxylate (**5c**).

## Sample Availability

Samples of the compounds are available from the authors.
