# Supplementary material for: Crystallography, Molecular Modeling, and COX-2 Inhibition Studies on Indolizine Derivatives
Source: Molecules. 2021 Jun 10;26(12):3550. doi: 10.3390/molecules26123550 (PMC8230391; doi:10.3390/molecules26123550)

Crystallography, Molecular Modelling and COX-2 Inhibition Studies on Indolizine Derivatives

Katharigatta N. Venugopala [1,2,*], Sandeep Chandrashekharappa [3,4*], Christophe Tratrat [1], Pran Kishore Deb [5], Rahul D. Nagdeve [6], Susanta K. Nayak [6], Mohamed A. Morsy [1,7], Pobitra Borah [8], Fawzi M. Mahomoodally [9], Raghu Prasad Mailavaram [10], Mahesh Attimarad [1], Bandar E. Aldhubiab [1], Nagaraja Sreeharsha [1,11], Anroop B. Nair [1], Osama I. Alwassil [12], Michelyne Haroun [1], Viresh Mohanlall [2], Pottathil Shinu[13], Rashmi Venugopala [14], Mahmoud Kandeel [15,16], Belakatte B. Nandeshwarappa[17] and Yasmine F. Ibrahim [7]

[1] Department of Pharmaceutical Sciences, College of Clinical Pharmacy, King Faisal University, Al-Ahsa 31982, Saudi Arabia; ctratrat@kfu.edu.sa (C.T.); momorsy@kfu.edu.sa (M.A.M.); mattimarad@kfu.edu.sa (M.A.); baldhubiab@kfu.edu.sa (B.E.A.); sharsha@kfu.edu.sa (N.S.); anair@kfu.edu.sa (A.B.N.); mharoun@kfu.edu.sa (M.H.)

[2] Department of Biotechnology and Food Technology, Durban University of Technology, Durban 4001, South Africa; vireshm@dut.ac.za (V.M.)

[3] Department of Medicinal Chemistry, National Institute of Pharmaceutical Education and Research (NIPER-R) Raebareli, Lucknow (UP)-226002, India; c.sandeep@niperraebareli.edu.in (S.C.)

[4] Institute for Stem Cell Science and Regenerative Medicine, NCBS, TIFR, GKVK, Bellary Road, Bangalore 560065, India; sandeepc@instem.res.in (S.C.)

[5] Faculty of Pharmacy, Philadelphia University, Amman 19392, Jordan; prankishore1@gmail.com (P.K.D.)

[6] Department of Chemistry, Visvesvaraya National Institute of Technology, Nagpur 440010, Maharashtra, India; rahulnagdeve3@gmail.com (R.D.N.); sknayak@chm.vnit.ac.in (S.K.N.)

[7] Department of Pharmacology, Faculty of Medicine, Minia University, El-Minia 61511, Egypt; yasmine.ibrahim@mu.edu.eg (Y.F.I.)

[8] Pratiksha Institute of Pharmaceutical Sciences, Chandrapur Road, Panikhaiti, Guwahati 781026, Assam, India; pobitrab.phe15@itbhu.ac.in (P.B.)

[9] Department of Health Sciences, Faculty of Science, University of Mauritius, Réduit 80835, Mauritius; f.mahomoodally@uom.ac.mu (F.M.M.)

[10] Department of Pharmaceutical Chemistry, Shri Vishnu College of Pharmacy, Vishnupur, Bhimavaram 534202, India; raghumrp@svcp.edu.in (R.P.M.)

[11] Department of Pharmaceutics, Vidya Siri College of Pharmacy, Off Sarjapura Road, Bangalore 560035, India

[12] Department of Pharmaceutical Sciences, College of Pharmacy, King Saud bin Abdulaziz University for health sciences, Riyadh 11481, Saudi Arabia; wassilo@ksau-hs.edu.sa (O.I.A.)

[13] Department of Biomedical Sciences, College of Clinical Pharmacy, King Faisal University, Al-Ahsa 31982, Saudi Arabia; spottathail@kfu.edu.sa (P.S.)

[14] Department of Public Health Medicine, University of KwaZulu-Natal, Howard College Campus, Durban 4001, South Africa; rashmivenugopala@gmail.com (R.V.)

[15] Department of Biomedical Sciences, College of Veterinary Medicine, King Faisal University, Al-Ahsa 31982, Saudi Arabia; mkandeel@kfu.edu.sa (M.K.)

[16] Department of Pharmacology, Faculty of Veterinary Medicine, Kafrelsheikh University, Kafrelsheikh 33516, Egypt

[17] Department of PG Studies and Research in Chemistry, Shivagangothri, Davangere University, Davangere, Karnataka 577 007, India; belakatte@davangereuniversity.ac.in (N.B.P.)

---

* Correspondence: kvenugopala@kfu.edu.sa; Tel.: +966-1358-98842 (K.N.V.); c.sandeep@niperraebareli.edu.in (S.C.); +91-94486-39413

Table of Contents

Figures	Contents	Page number
	General Synthetic Procedure for 4-Methoxy-1-(2-(substituted phenyl)-2-oxoethyl)pyridinium Bromide (**3a-e**)	3
	Synthetic Procedure for the Synthesis of ethyl 3-(substituted benzoyl)-2-ethyl-7-methoxyindolizine-1-carboxylate (**5e**)	3
S1	FT-IR of diethyl 3-(4-cyanobenzoyl)-7-methoxyindolizine-1,2-dicarboxylate (**5a**)	6
S2	^{1}H-NMR of diethyl 3-(4-cyanobenzoyl)-7-methoxyindolizine-1,2-dicarboxylate (**5a**)	7
S3	^{13}C-NMR of diethyl 3-(4-cyanobenzoyl)-7-methoxyindolizine-1,2-dicarboxylate (**5a**)	8
S4	FT-IR of diethyl 3-(4-fluorobenzoyl)-7-methoxyindolizine-1,2-dicarboxylate (**5b**)	9
S5	^{1}H-NMR of diethyl 3-(4-fluorobenzoyl)-7-methoxyindolizine-1,2-dicarboxylate (**5b**)	10
S6	^{13}C-NMR of diethyl 3-(4-fluorobenzoyl)-7-methoxyindolizine-1,2-dicarboxylate (**5b**)	11
S7	FT-IR of diethyl 3-(4-bromobenzoyl)-7-methoxyindolizine-1,2-dicarboxylate (**5c**)	12
S8	^{1}H-NMR of diethyl 3-(4-bromobenzoyl)-7-methoxyindolizine-1,2-dicarboxylate (**5c**)	13
S9	^{13}C-NMR of diethyl 3-(4-bromobenzoyl)-7-methoxyindolizine-1,2-dicarboxylate (**5c**)	14
S10	FT-IR of diethyl 7-methoxy-3-(3-methoxybenzoyl)indolizine-1,2-dicarboxylate (**5d**)	15
S11	^{1}H-NMR of diethyl 7-methoxy-3-(3-methoxybenzoyl)indolizine-1,2-dicarboxylate (**5d**)	16
S12	^{13}C-NMR of diethyl 7-methoxy-3-(3-methoxybenzoyl)indolizine-1,2-dicarboxylate (**5d**)	17
S13	FT-IR of ethyl 3-(4-bromobenzoyl)-2-ethyl-7-methoxyindolizine-1-carboxylate (**5e**)	18
S14	^{1}H-NMR of ethyl 3-(4-bromobenzoyl)-2-ethyl-7-methoxyindolizine-1-carboxylate (**5e**)	19
S15	^{13}C-NMR of ethyl 3-(4-bromobenzoyl)-2-ethyl-7-methoxyindolizine-1-carboxylate (**5e**)	20
S16	checkCIF/PLATON report of diethyl 3-(4-bromobenzoyl)-7-methoxyindolizine-1,2-dicarboxylate (**5c**)	21-24

1. General Synthetic Procedure for 4-Methoxy-1-(2-(substituted phenyl)-2-oxoethyl)pyridinium Bromide (3a-e)

To a solution of 4-methoxypyridine (**1**) (0.0091 mol, 1 g) in dry acetone solvent (10 mL), substituted-phenacylbromide (0.0091 mol, 2.03 g) was added and agitated at room temperature for 5 h. The completion of reaction was observed on thin-layer chromatography (TLC). A product obtained was separated, filtered, and desiccated under vacuum to yield a 92-99% of 1-(2-(substituted phenyl)2-oxoethyl)-4-methoxypyridinium bromides.

2. General Procedure for the Synthesis of Ethyl 7-acetyl-3-(4-substitutredbenzoyl)-2-substitutedindolizine-1-carboxylate (5a-e)

To a stirred solution of 1-(2-(substituted phenyl)-2-oxoethyl)-4-methoxypyridinium bromide (1 g), in dry dimethylformamide, diethyl but-2-ynedioate (4) (0.0.512 g) and K_2CO_3 (0.831 g) were added. It was stirred at room temperature for 30 min. The completion of the reaction was monitored on TLC. After completion of the reaction, the solvent was evaporated under reduced pressure and diluted with ethyl acetate. The organic layer was washed with water, brine, and dried with sodium sulfate. The crude compound was purified by column chromatography to afford a 72% yield of compound 5a. Similarly, other compounds 5b-5e of the series were prepared, purified, and physicochemical characteristics are tabulated in Table 1.

2.1. Diethyl 3-(4-cyanobenzoyl)-7-methoxyindolizine-1,2-dicarboxylate (**5a**)

Appearance: Light brown compound. FT-IR (KBR neat cm^{-1}) 2987, 2229, 1737, 1693, 1647, 1596. ^{1}H-NMR (400 MHz $CDCl_3$) δ = 9.55-9.53 (d, J = 7.2Hz, 1H), 7.78 (s, 1H), 7.61-7.55 (m, 4H), 6.82-6.79 (m, 1H), 4.36-4.31 (q, J = 7.2Hz, 2H), 3.99 (s, 3H), 3.76-3.70 (q, J = 7.2Hz, 2H), 1.36-1.33 (t, J = 7.2Hz, 3H), 1.14-1.10 (t, J = 7.2Hz, 3H). ^{13}C-NMR (100 MHz $CDCl_3$) δ = 184.89, 164.98, 163.21, 160.09, 141.57, 138.57, 132.53, 131.21, 130.23, 130.05, 126.28, 119.40, 110.24, 102.64, 97.66, 61.77, 60.28, 55.83, 14.23, 13.60. LC-MS (ESI

positive) *m/z*: $(M+H)^+$: 421.2. Analytical calculated for $C_{23}H_{20}N_2O_6$: C, 65.71, H, 4.79, N, 6.66; found; C, 65.75, H, 4.77, N, 6.62.

2.2. Diethyl 3-(4-fluorobenzoyl)-7-methoxyindolizine-1,2-dicarboxylate (**5b**)

Appearance: Yellow amorphous compound. FT-IR (KBR neat cm^{-1}) 2981, 1737, 1699, 1647, 1607. ^1H-NMR (400 MHz $CDCl_3$) δ = 9.50-9.48 (d, *J* = 7.2Hz, 1H), 7.77-7.71 (m, 3H), 7.15-7.11 (m, 2H), 6.81-6.78 (m, 1H), 4.36-4.31 (q, *J* = 7.2Hz, 2H), 3.98 (s, 3H), 3.75-3.69 (q, *J* = 7.2Hz, 2H), 1.36-1.33 (t, *J* = 7.2Hz, 3H), 1.13-1.10 (t, *J* = 7.2Hz, 3H). ^{13}C-NMR (100 MHz $CDCl_3$) δ = 184.77, 166.11, 165.02, 163.60, 163.27, 159.96, 141.47, 136.04, 136.00, 132.25, 131.23, 131.14, 129.96, 119.58, 115.59, 114.97, 110.15, 102.44, 97.59, 61.68, 60.25, 55.81, 14.23, 13.62. LC-MS (ESI positive) *m/z*: $(M+H)^+$: 414.2. Analytical calculated for $C_{22}H_{20}FNO_6$: C, 63.92, H, 4.88, N, 3.39; found; C, 63.98, H, 4.85, N, 3.40.

2.3. Diethyl 3-(4-bromobenzoyl)-7-methoxyindolizine-1,2-dicarboxylate (**5c**)

Appearance: Light brown crystalline compound. FT-IR (KBR neat cm^{-1}): 1739, 1691, 1643, 1606. ^1H-NMR (400 MHz $CDCl_3$) δ = 9.55-9.53 (d, *J* = 7.2Hz, 1H), 7.78-7.77 (m, 1H), 7.61-7.55 (m, 4H), 6.82-6.79 (m, 1H), 4.36-4.31 (q, *J* = 7.2Hz, 2H), 3.99 (s, 3H), 3.76-3.70 (q, *J* = 7.2Hz, 2H), 1.36-1.33 (t, *J* = 7.2Hz, 3H), 1.14-1.10 (t, *J* = 7.2Hz, 3H). ^{13}C-NMR (100 MHz $CDCl_3$) δ = 184.89, 164.98, 163.21, 160.09, 141.57, 138.57, 132.53, 131.21, 130.23, 130.05, 126.28, 119.40, 110.24, 102.64, 97.66, 61.77, 60.28, 55.83, 18.44, 14.23, 13.60. LC-MS (ESI, Positive): *m/z* 474.12 $(M)^+$, 476.2 $(M+H)^+$. Analytical calculated for $C_{22}H_{20}BrNO_6$; C, 55.71; H, 4.25; N, 2.95; found: C, 55.74: H, 4.22: N, 2.79.

2.4. Diethyl 7-methoxy-3-(3-methoxybenzoyl)indolizine-1,2-dicarboxylate (**5d**)

Appearance: Light yellow crystalline compound. FT-IR (KBR neat cm^{-1}) 2981, 1738, 1693, 1647, 1608. ^1H-NMR (400 MHz $CDCl_3$) δ = 9.56-9.54 (d, *J* = 7.2Hz, 1H), 7.78 (s, 1H), 7.38-7.36 (m, 1H), 7.29-7.28 (m, 1H), 7.21 (s, 1H), 7.10-7.08 (m, 1H), 6.80-6.78 (m, 1H), 4.35-4.30 (q, *J* = 7.2Hz, 2H), 3.99 (s, 3H), 3.81 (s, 3H), 3.73-3.68 (q, *J* = 7.2Hz, 2H), 1.36-1.32 (t, *J* = 7.2Hz, 3H), 1.11-1.08 (t, *J* = 7.2Hz, 3H). ^{13}C-NMR (100 MHz $CDCl_3$) δ = 185.90, 165.08, 163.35, 159.91, 159.09, 141.46, 141.00, 132.45, 130.10, 129.14, 121.14,

119.73, 118.38, 112.99, 110.08, 102.45, 97.57, 61.63, 60.21, 55.80, 55.36, 14.22, 13.54. LC-MS (ESI positive) *m/z* 426.14 $(M+H)^+$. Analytical calculated for $C_{23}H_{23}NO_7$: C, 64.93, H, 5.45, N, 3,29; found; C, 64.95, H, 5.42, N, 3.32.

2.5. Ethyl 3-(4-bromobenzoyl)-2-ethyl-7-methoxyindolizine-1-carboxylate (**5e**)

Appearance: Light yellow crystalline compound. FT-IR (KBR neat cm^{-1}): 1699, 1668, 1639, 1602. ^1H-NMR (400 MHz $CDCl_3$) δ = 9.18-9.17 (d, *J* = 7.2Hz, 1H), 7.69-7.60 (m, 3H), 7.10-7.05 (m, 2H), 6.57-6.54 (m, 1H), 4.33-4.28 (q, *J* = 7.2Hz, 2H), 3.86 (s, 3H), 2.60-2.55 (q, *J* = 7.2Hz, 2H), 1.36-1.31 (t, *J* = 7.2Hz, 3H), 0.93-0.89 (t, *J* = 7.2Hz, 3H). ^{13}C-NMR (100 MHz $CDCl_3$) δ = 186.00, 166.07, 165.01, 163.56, 159.37, 144.79, 142.77, 137.58, 137.55, 130.95, 130.86, 129.61, 121.61, 121.10, 115.64, 115.42, 108.20, 102.82, 97.57, 59.64, 55.57, 20.06, 15.99, 14.41. Analytical calculated for $C_{21}H_{20}BrNO_4$: C, 58.62; H, 4.68; N, 3.26; Found: C, 58.69: H, 4.52: N, 3.24.

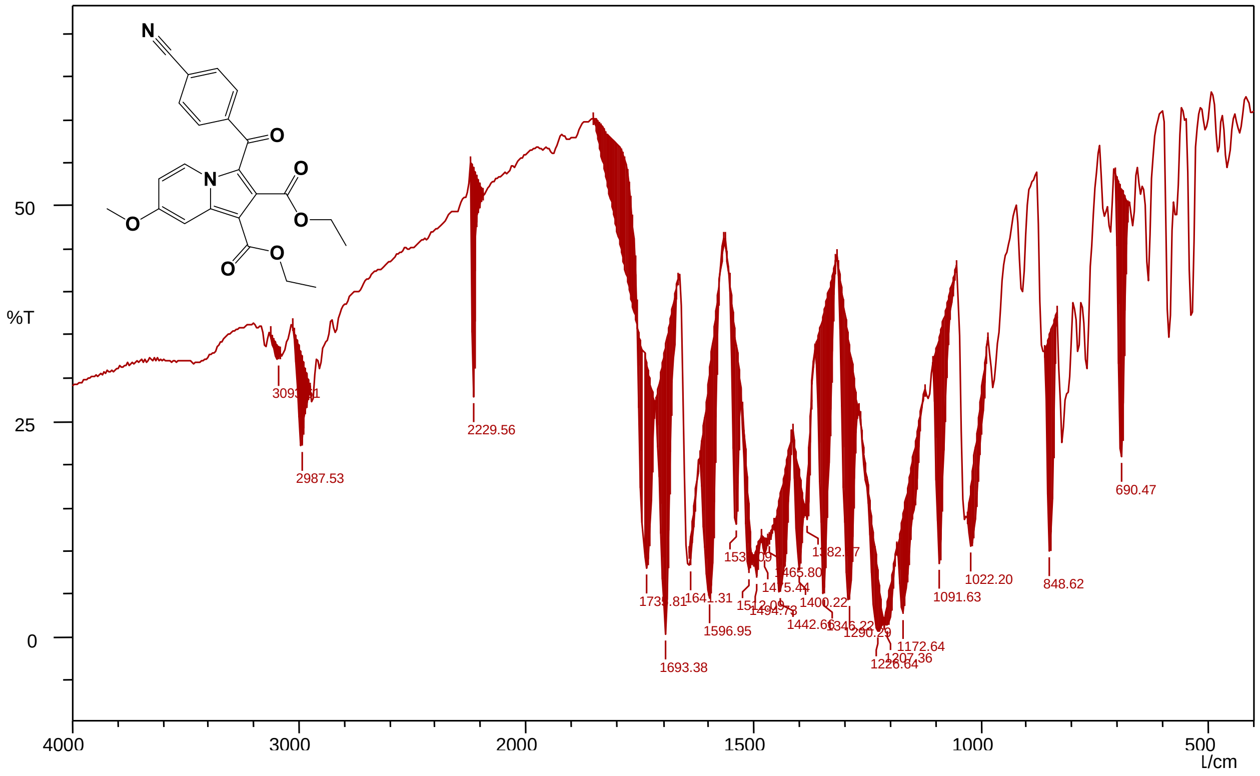

Figure S1. FT-IR of diethyl 3-(4-cyanobenzoyl)-7-methoxyindolizine-1,2-dicarboxylate (**5a**).

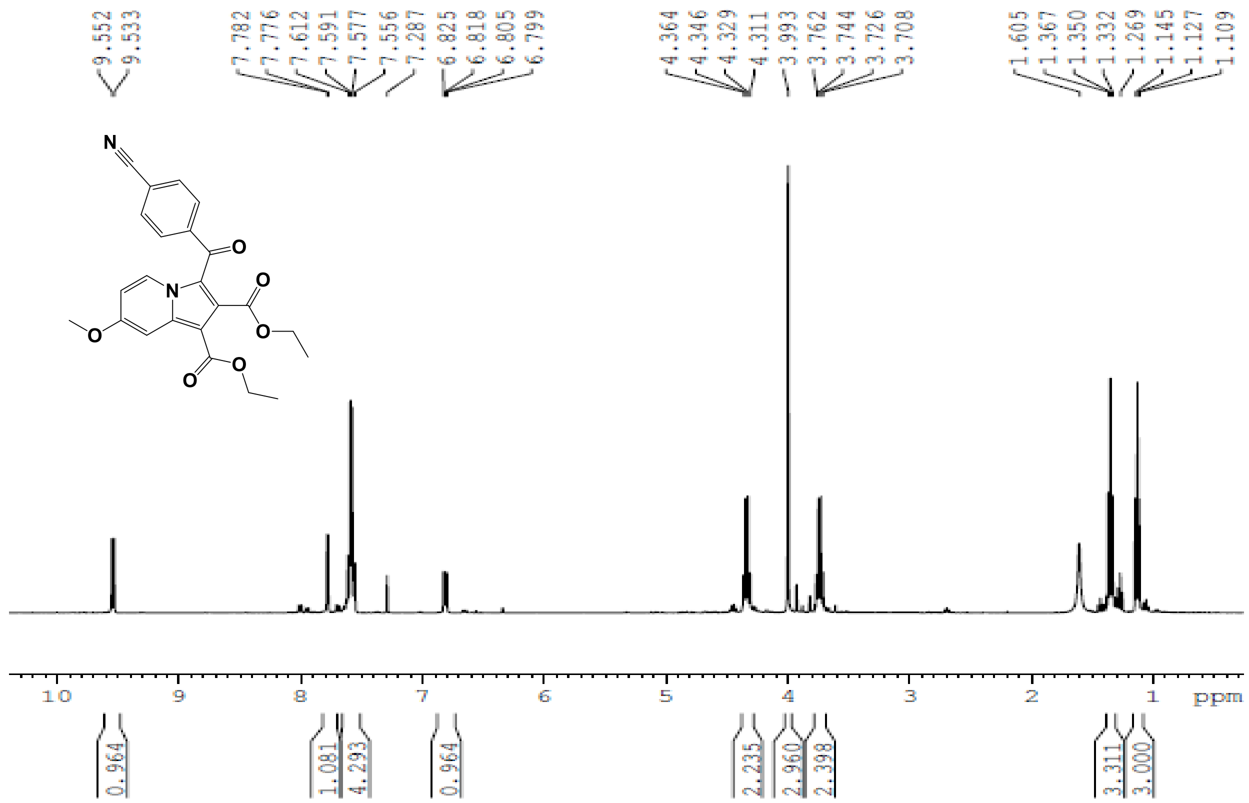

Figure S2. ^{1}H-NMR of diethyl 3-(4-cyanobenzoyl)-7-methoxyindolizine-1,2-dicarboxylate (**5a**).

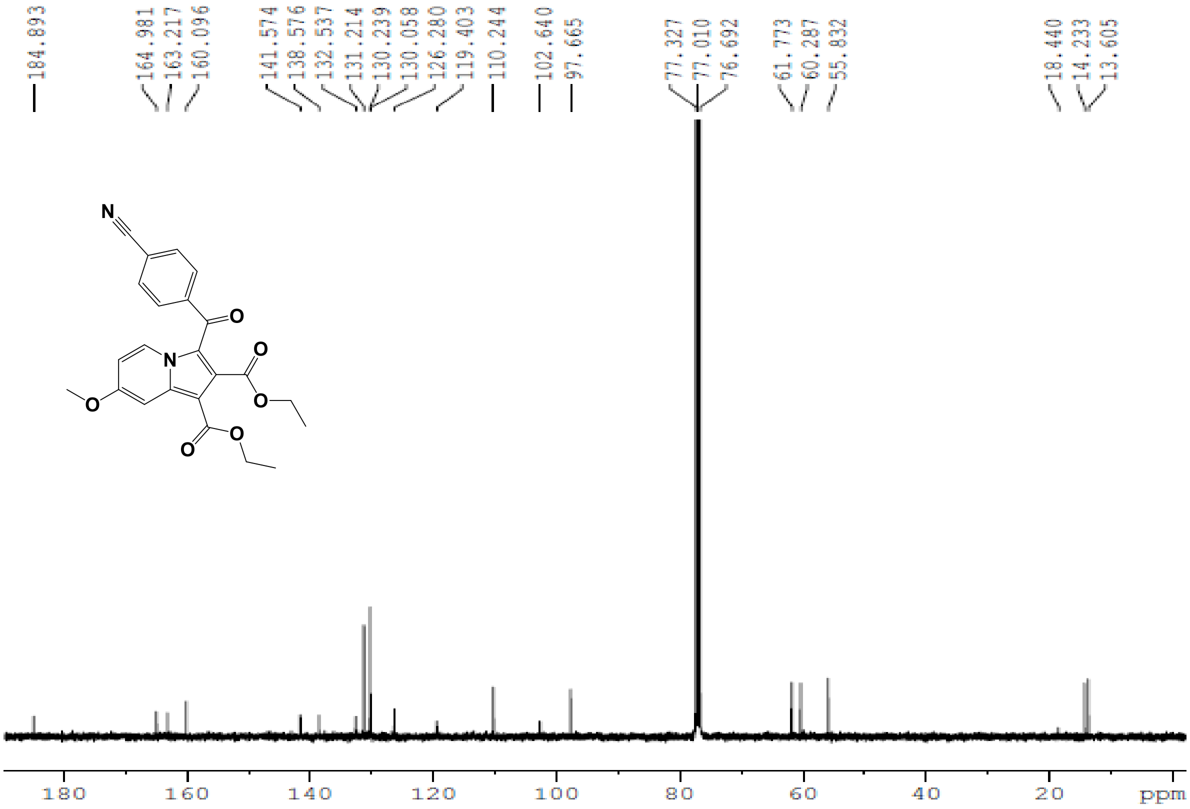

Figure S3. ^{13}C-NMR of diethyl 3-(4-cyanobenzoyl)-7-methoxyindolizine-1,2-dicarboxylate (**5a**).

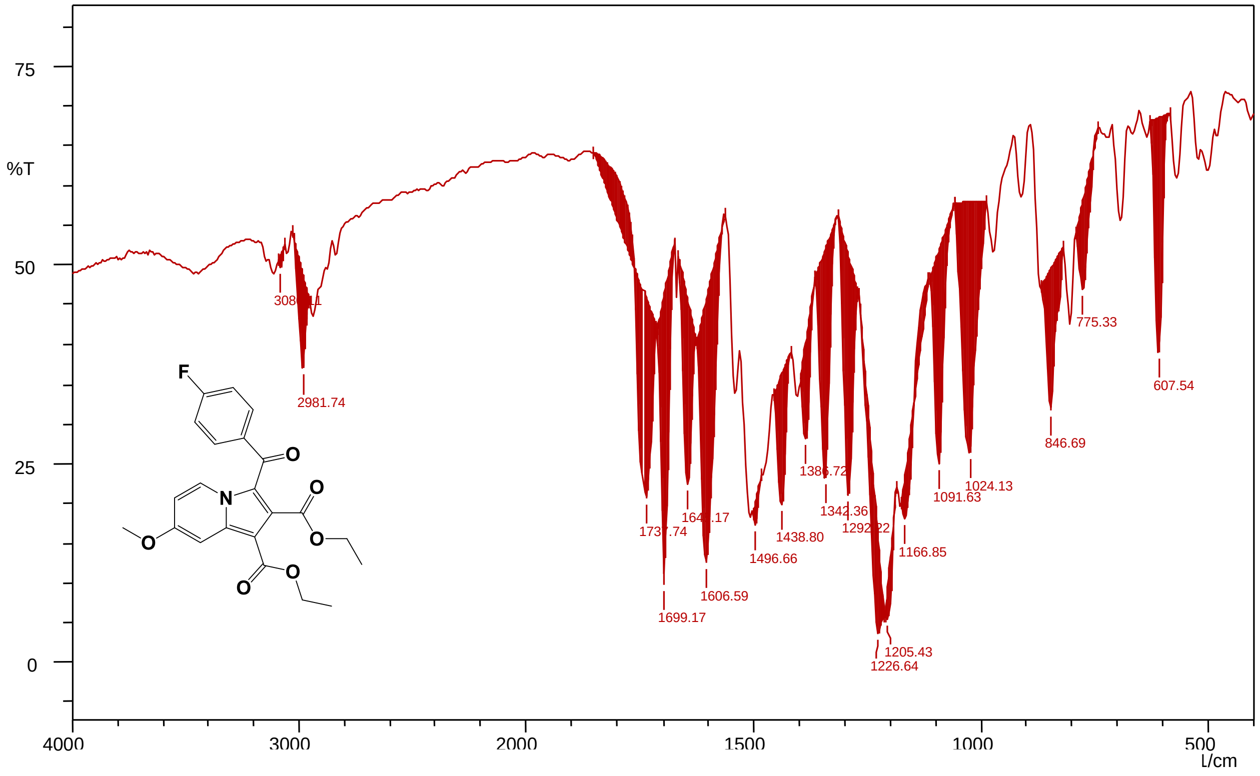

Figure S4. FT-IR of diethyl 3-(4-fluorobenzoyl)-7-methoxyindolizine-1,2-dicarboxylate (**5b**).

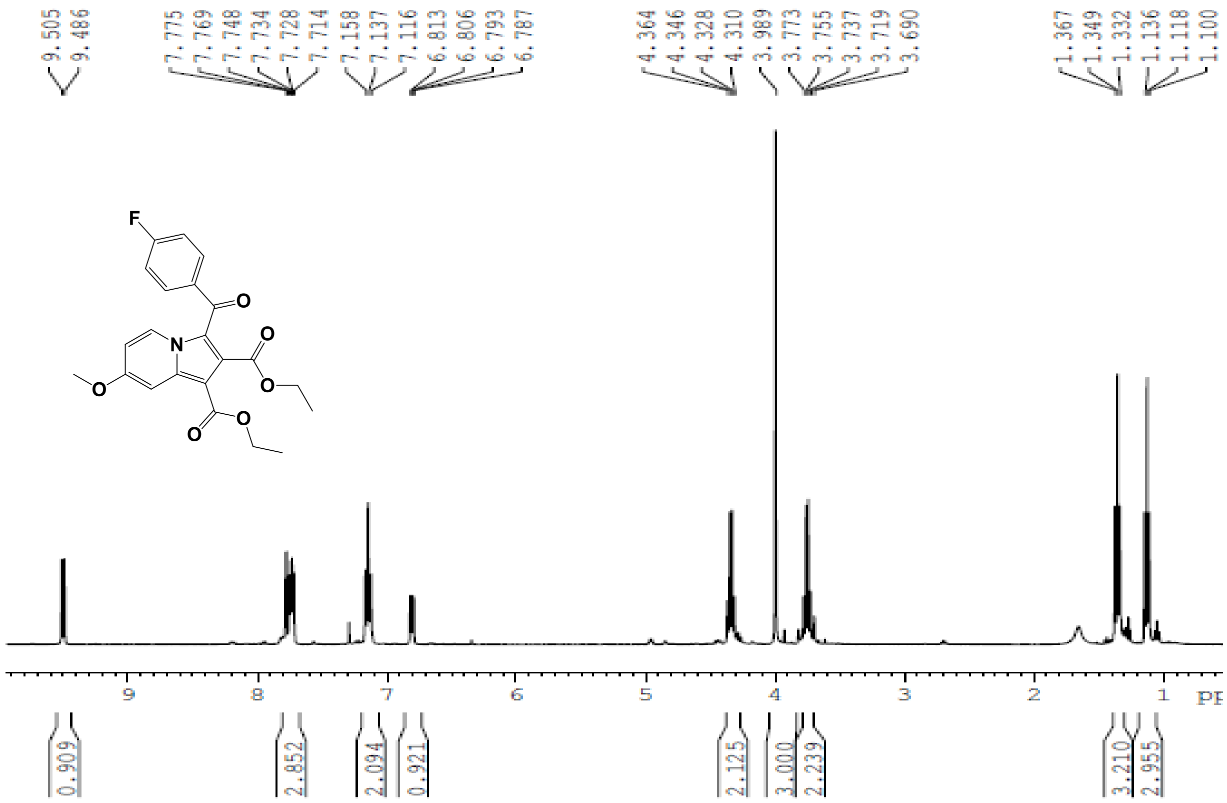

Figure S5. ^{1}H-NMR of diethyl 3-(4-fluorobenzoyl)-7-methoxyindolizine-1,2-dicarboxylate (**5b**).

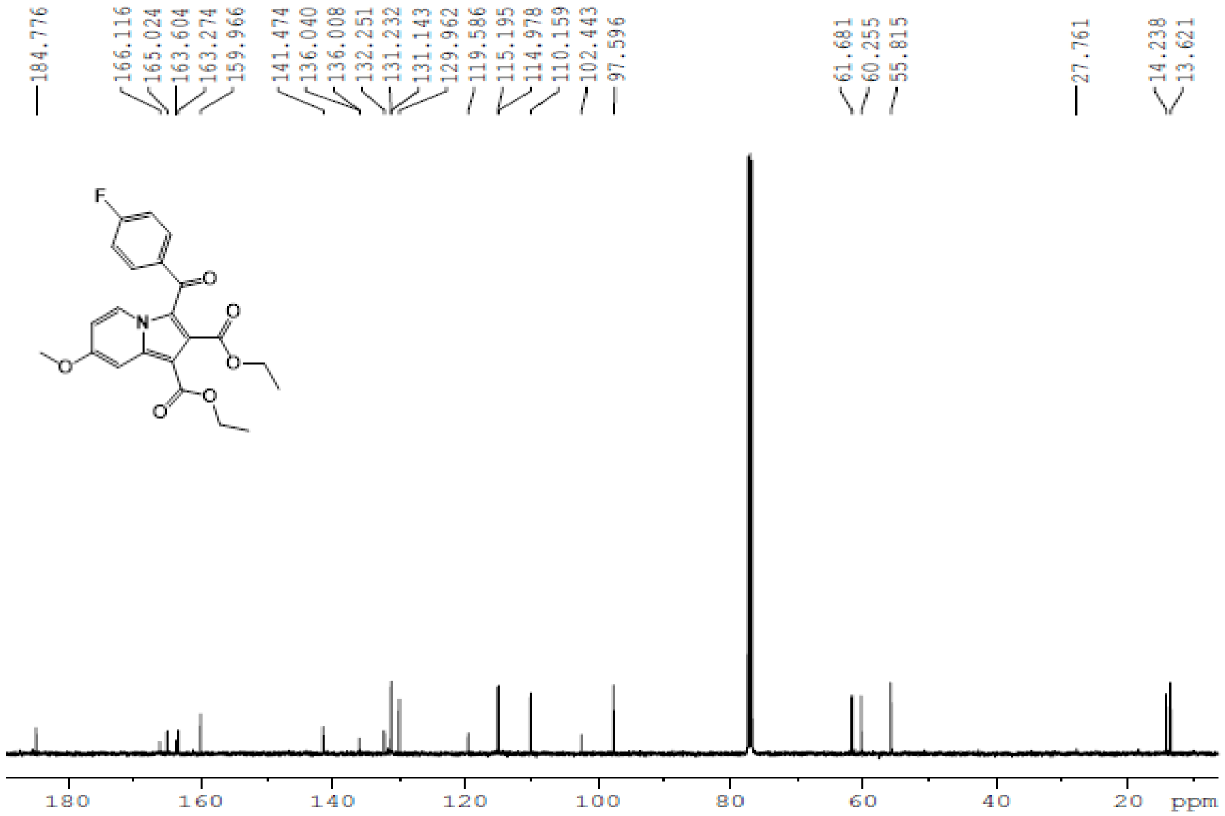

Figure S6. ^{13}C-NMR of diethyl 3-(4-fluorobenzoyl)-7-methoxyindolizine-1,2-dicarboxylate (**5b**).

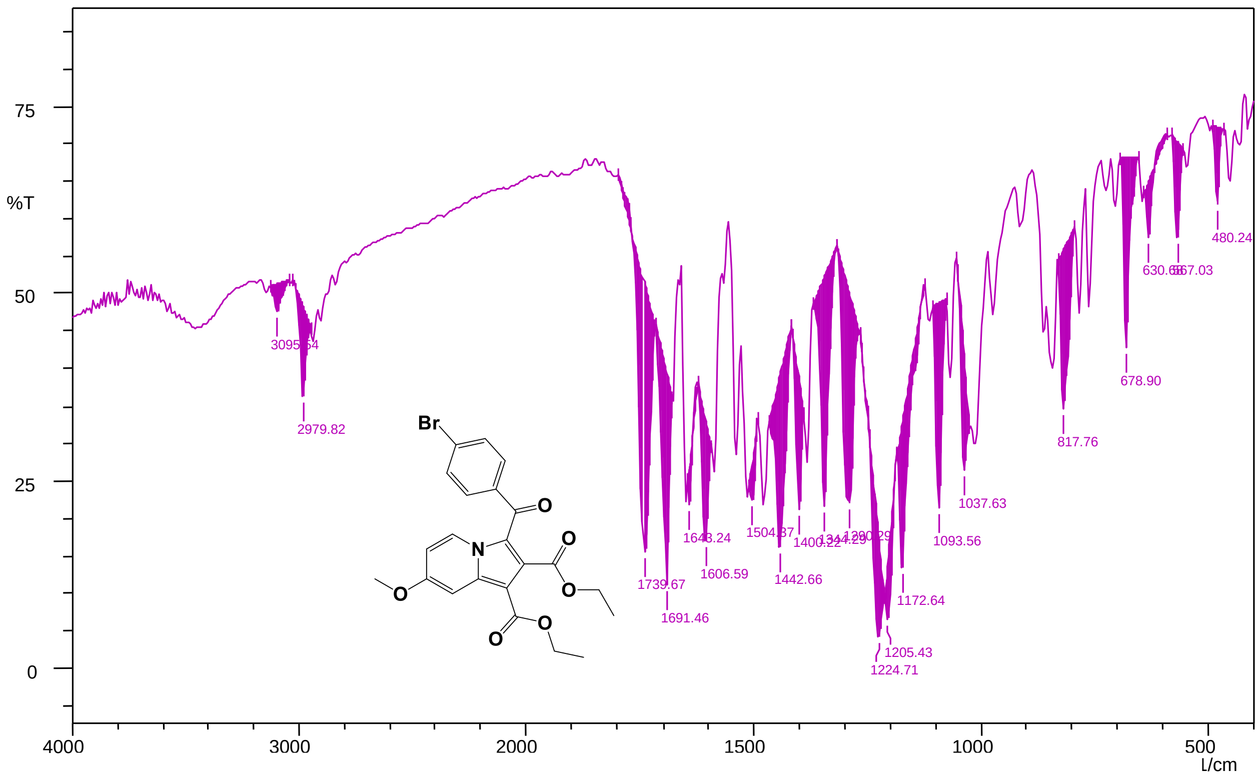

Figure S7. FT-IR of diethyl 3-(4-bromobenzoyl)-7-methoxyindolizine-1,2-dicarboxylate (**5c**).

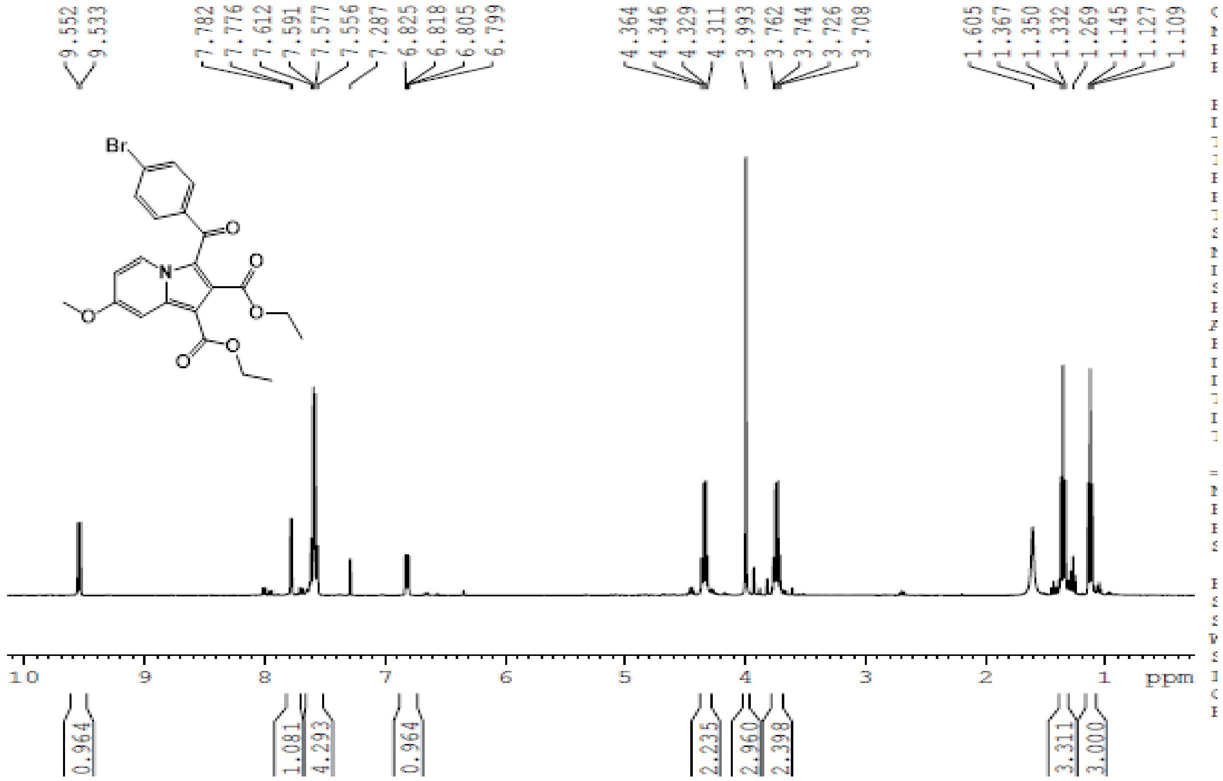

Figure S8. ^{1}H-NMR of diethyl 3-(4-bromobenzoyl)-7-methoxyindolizine-1,2-dicarboxylate (**5c**).

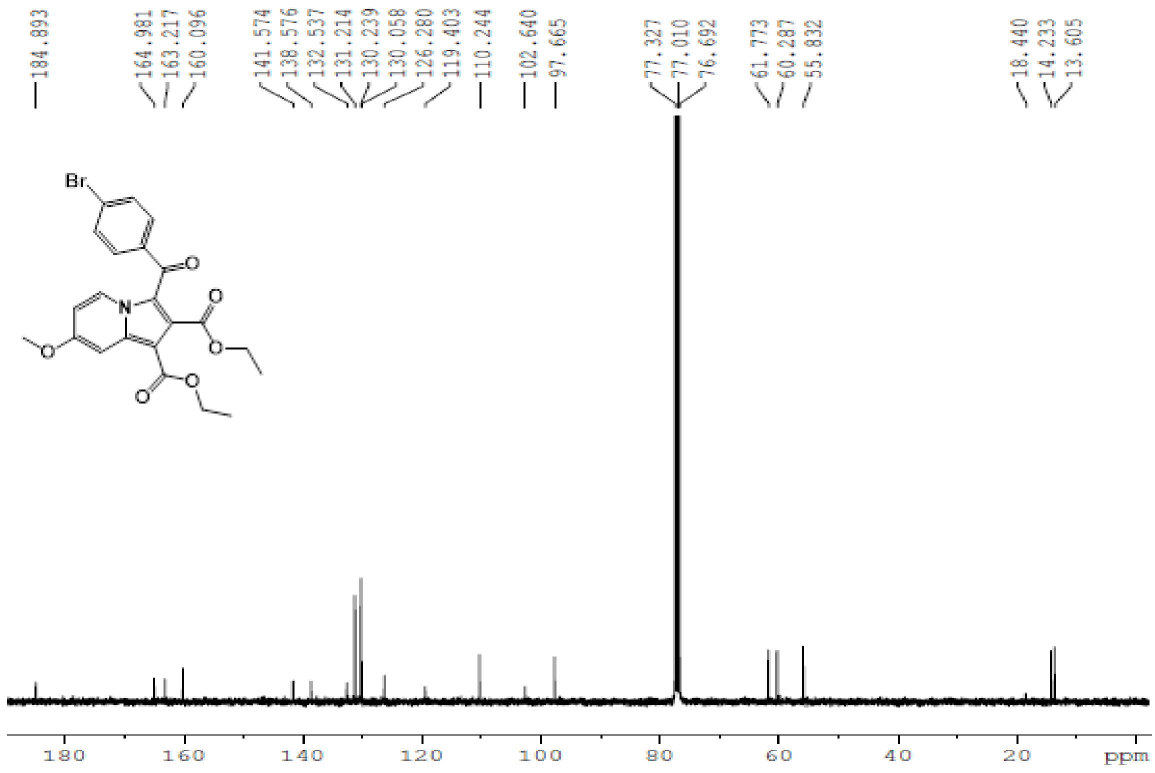

Figure S9. ^{13}C-NMR of diethyl 3-(4-bromobenzoyl)-7-methoxyindolizine-1,2-dicarboxylate (**5c**).

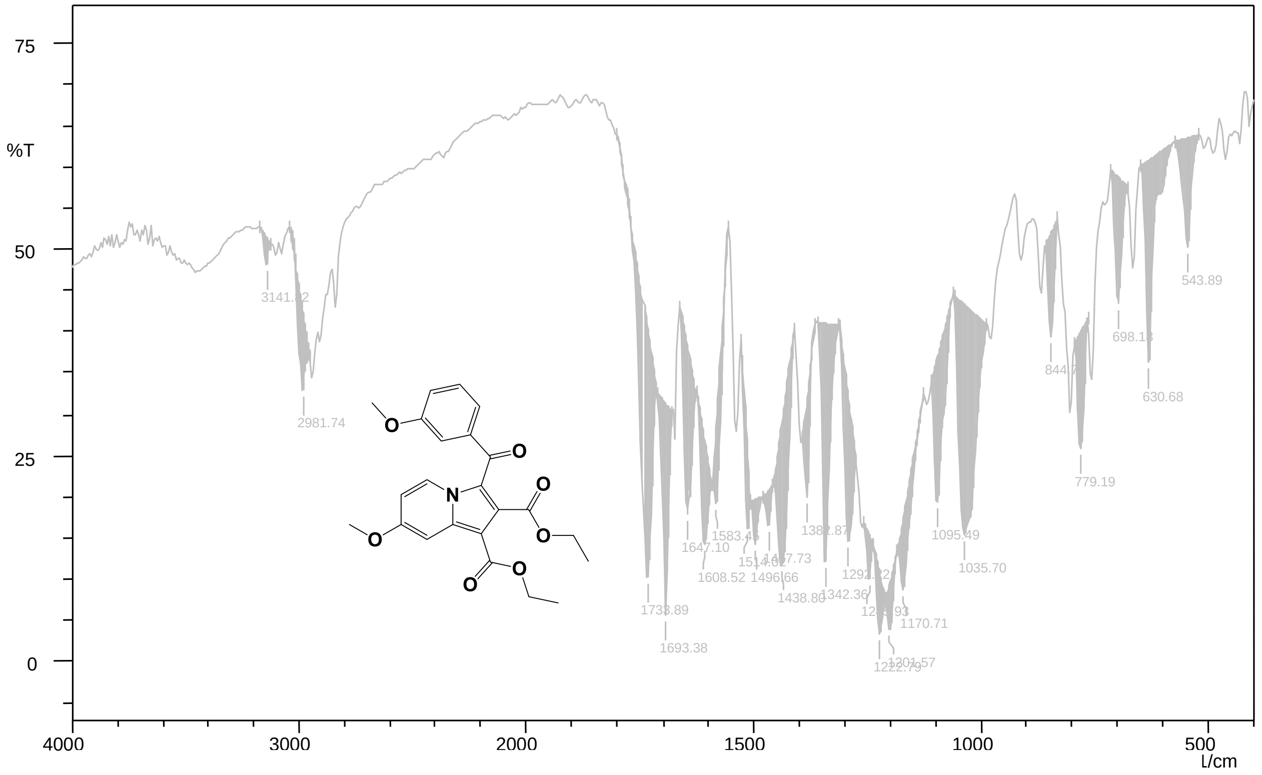

Figure S10. FT-IR of diethyl 7-methoxy-3-(3-methoxybenzoyl)indolizine-1,2-dicarboxylate (**5d**).

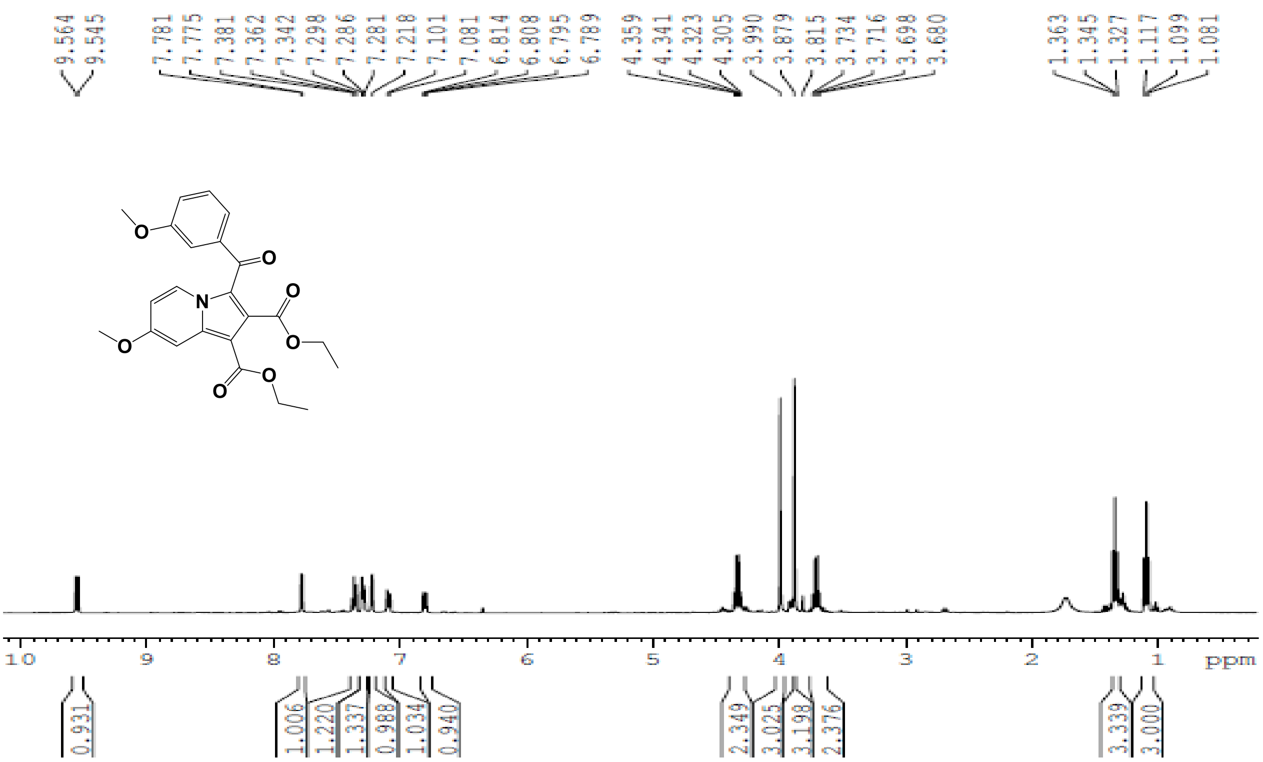

Figure S11. ^{1}H-NMR of diethyl 7-methoxy-3-(3-methoxybenzoyl)indolizine-1,2-dicarboxylate (**5d**).

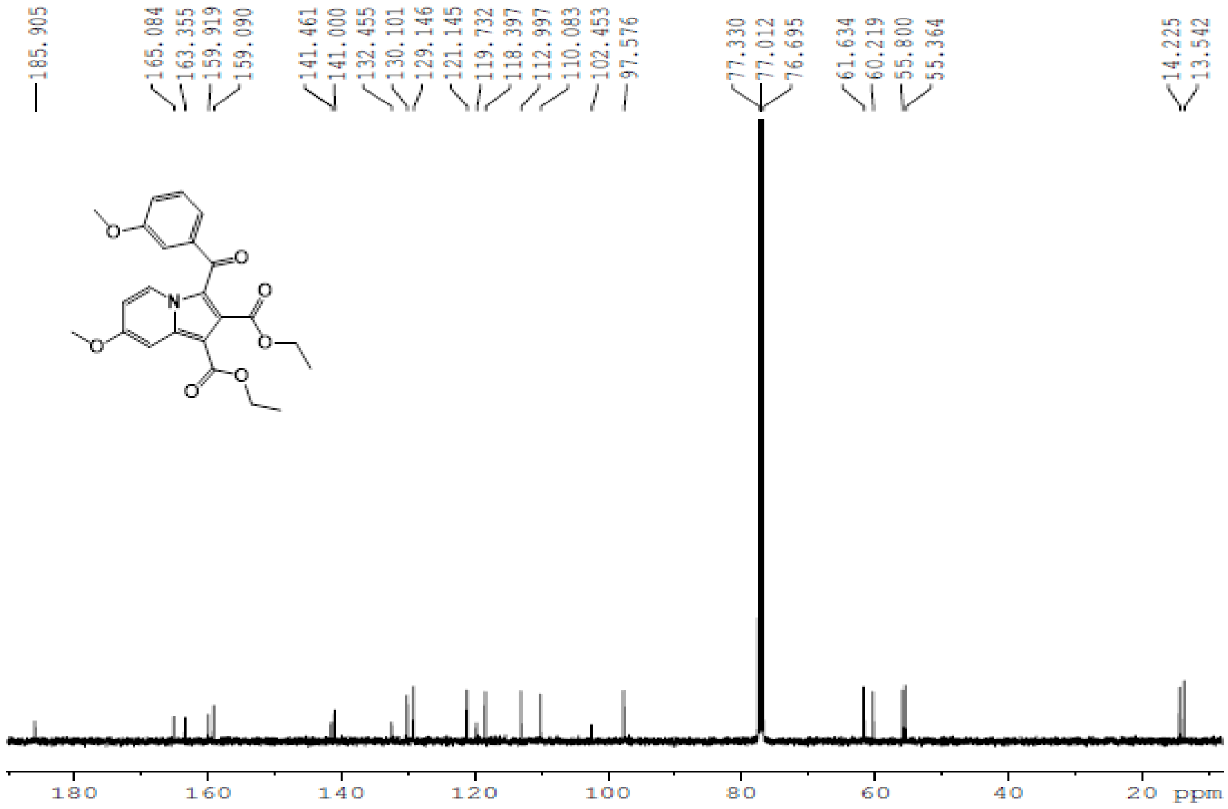

Figure S12. ^{13}C-NMR of diethyl 7-methoxy-3-(3-methoxybenzoyl)indolizine-1,2-dicarboxylate (**5d**).

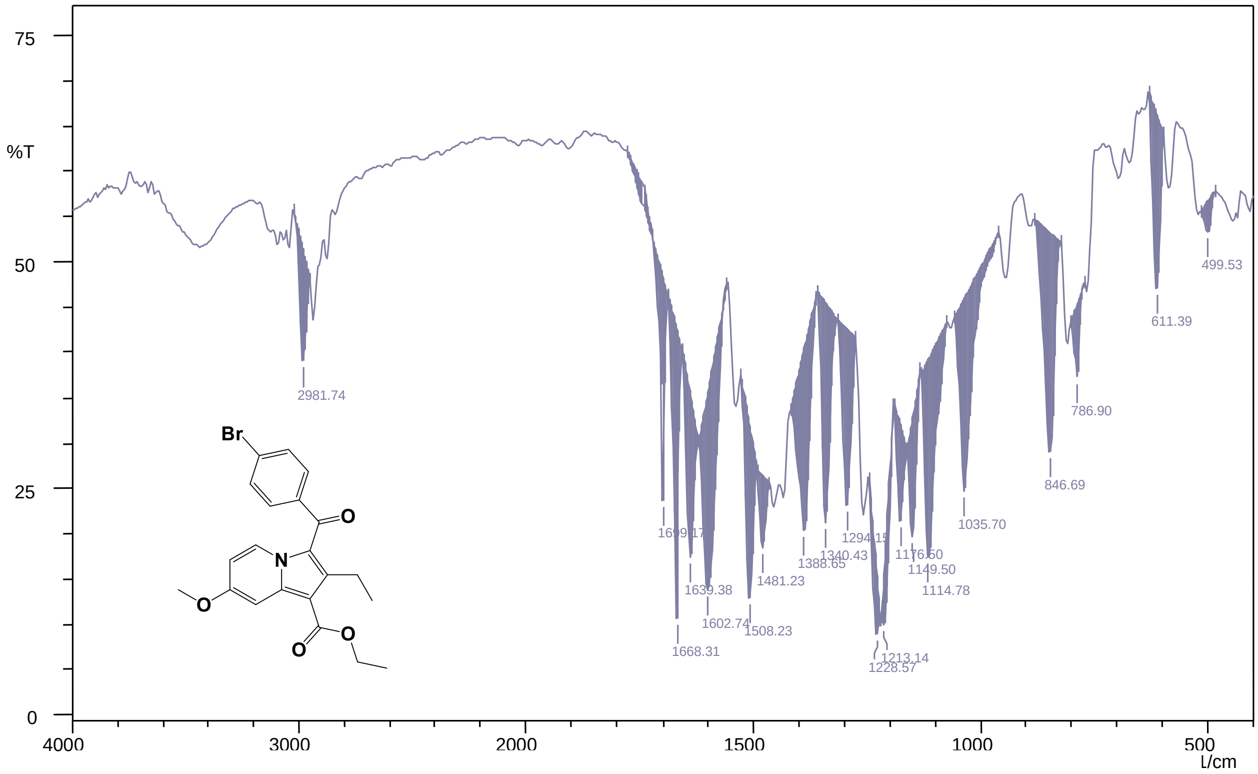

Figure S13. FT-IR of ethyl 3-(4-bromobenzoyl)-2-ethyl-7-methoxyindolizine-1-carboxylate (**5e**).

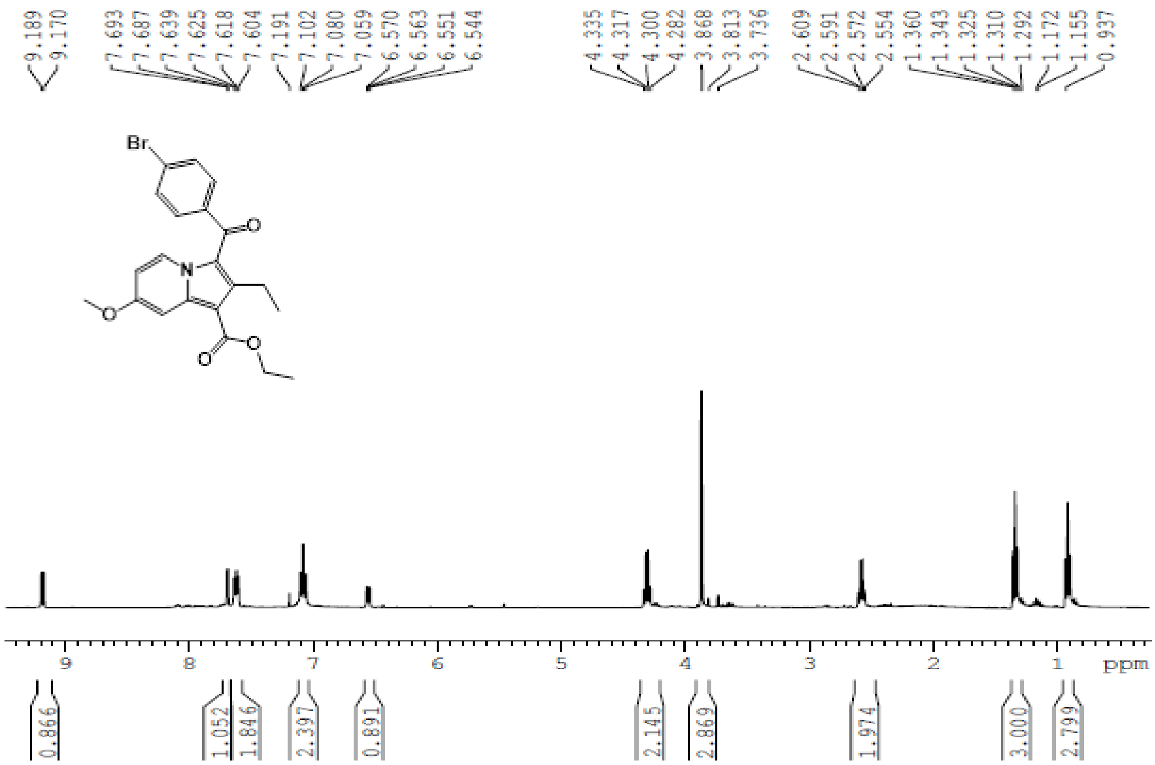

Figure S14. ^{1}H-NMR of ethyl 3-(4-bromobenzoyl)-2-ethyl-7-methoxyindolizine-1-carboxylate (**5e**).

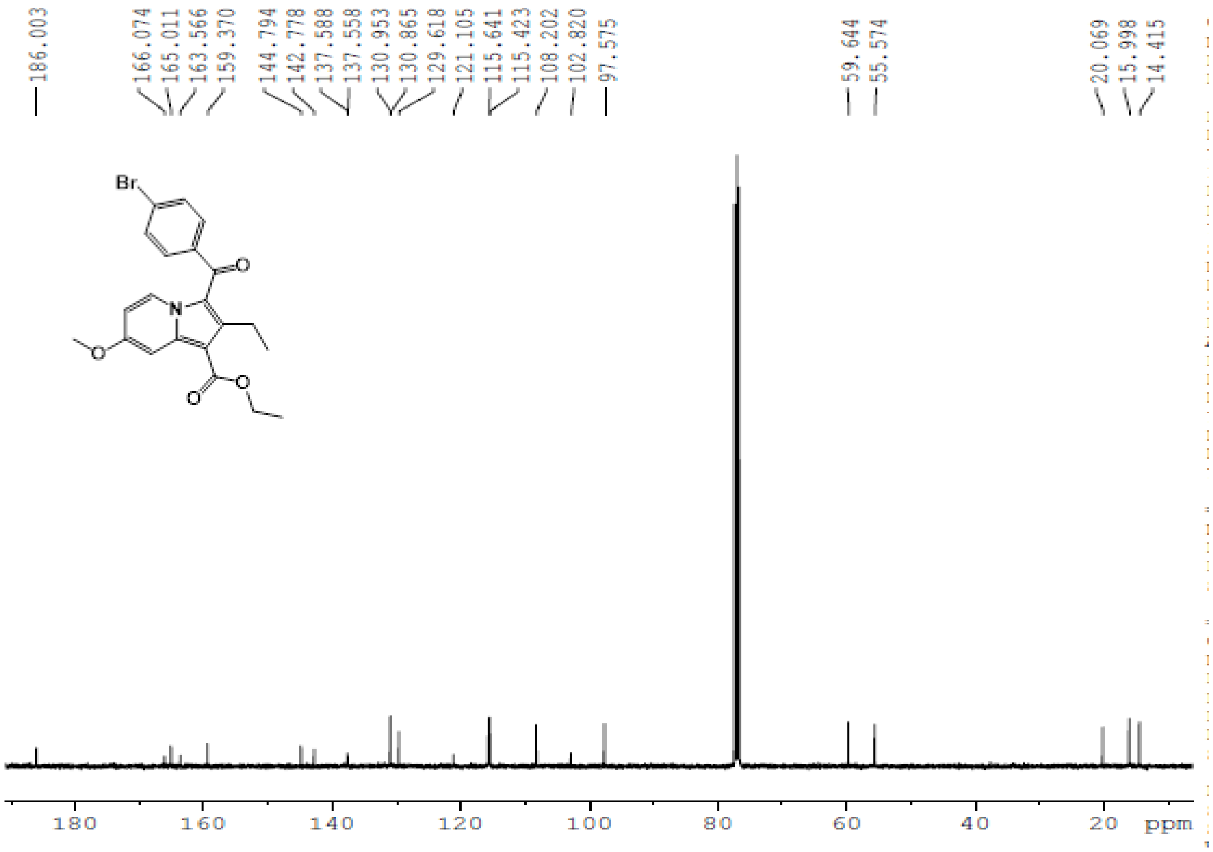

Figure S15. ^{13}C-NMR of ethyl 3-(4-bromobenzoyl)-2-ethyl-7-methoxyindolizine-1-carboxylate (**5e**).

checkCIF/PLATON report of diethyl 3-(4-bromobenzoyl)-7-methoxyindolizine-1,2-dicarboxylate (**5c**)

checkCIF/PLATON report

Structure factors have been supplied for datablock(s) 2j

THIS REPORT IS FOR GUIDANCE ONLY. IF USED AS PART OF A REVIEW PROCEDURE FOR PUBLICATION, IT SHOULD NOT REPLACE THE EXPERTISE OF AN EXPERIENCED CRYSTALLOGRAPHIC REFEREE.

No syntax errors found. CIF dictionary Interpreting this report

Datablock: 2j

```
Bond precision:   C-C = 0.0031 A          Wavelength=0.71073

Cell:             a=12.0497(6)    b=17.8324(10)      c=19.6052(11)
                  alpha=90        beta=100.372(1)    gamma=90
Temperature:      173 K

                  Calculated                 Reported
Volume            4143.8(4)                  4143.8(4)
Space group       P 21/n                     P 21/n
Hall group        -P 2yn                     -P 2yn
Moiety formula    C22 H20 Br N O6            C22 H20 Br N O6
Sum formula       C22 H20 Br N O6            C22 H20 Br N O6
Mr                474.29                     474.30
Dx,g cm-3         1.520                      1.521
Z                 8                          8
Mu (mm-1)         2.023                      2.023
F000              1936.0                     1936.0
F000'             1934.65
h,k,lmax          16,23,26                   16,23,26
Nref              10288                      10282
Tmin,Tmax         0.550,0.591                0.837,1.000
Tmin'             0.540

Correction method= # Reported T Limits: Tmin=0.837 Tmax=1.000
AbsCorr = MULTI-SCAN

Data completeness= 0.999          Theta(max)= 28.293

R(reflections)= 0.0410( 6833)     wR2(reflections)= 0.1000( 10282)

S = 1.024                 Npar= 576
```

The following ALERTS were generated. Each ALERT has the format
test-name_ALERT_alert-type_alert-level.
Click on the hyperlinks for more details of the test.

```
 Alert level C
PLAT213_ALERT_2_C Atom O3C            has ADP max/min Ratio .....        3.5 prolat
PLAT220_ALERT_2_C NonSolvent   Resd 1  C   Ueq(max)/Ueq(min) Range       3.9 Ratio
PLAT222_ALERT_3_C NonSolvent Resd 1  H   Uiso(max)/Uiso(min) Range       4.3 Ratio
PLAT911_ALERT_3_C Missing FCF Refl Between Thmin & STh/L=    0.600         5 Report
```

```
 Alert level G
PLAT002_ALERT_2_G Number of Distance or Angle Restraints on AtSite         7 Note
PLAT003_ALERT_2_G Number of Uiso or Uij Restrained non-H Atoms ...         6 Report
PLAT176_ALERT_4_G The CIF-Embedded .res File Contains SADI Records         3 Report
PLAT177_ALERT_4_G The CIF-Embedded .res File Contains DELU Records         1 Report
PLAT178_ALERT_4_G The CIF-Embedded .res File Contains SIMU Records         1 Report
PLAT230_ALERT_2_G Hirshfeld Test Diff for    O3C      --C8B      .       6.0 s.u.
PLAT301_ALERT_3_G Main Residue  Disorder .............(Resd  1  )        10% Note
PLAT720_ALERT_4_G Number of Unusual/Non-Standard Labels .........         12 Note
PLAT860_ALERT_3_G Number of Least-Squares Restraints ............         75 Note
PLAT912_ALERT_4_G Missing # of FCF Reflections Above STh/L=  0.600         1 Note
PLAT933_ALERT_2_G Number of OMIT Records in Embedded .res File ...         5 Note
PLAT941_ALERT_3_G Average HKL Measurement Multiplicity ..........        4.7 Low
PLAT978_ALERT_2_G Number C-C Bonds with Positive Residual Density.         7 Info
PLAT992_ALERT_5_G Repd & Actual _reflns_number_gt Values Differ by         2 Check
```

```
   0 ALERT level A = Most likely a serious problem - resolve or explain
   0 ALERT level B = A potentially serious problem, consider carefully
   4 ALERT level C = Check. Ensure it is not caused by an omission or oversight
  14 ALERT level G = General information/check it is not something unexpected

   0 ALERT type 1 CIF construction/syntax error, inconsistent or missing data
   7 ALERT type 2 Indicator that the structure model may be wrong or deficient
   5 ALERT type 3 Indicator that the structure quality may be low
   5 ALERT type 4 Improvement, methodology, query or suggestion
   1 ALERT type 5 Informative message, check
```

It is advisable to attempt to resolve as many as possible of the alerts in all categories. Often the minor alerts point to easily fixed oversights, errors and omissions in your CIF or refinement strategy, so attention to these fine details can be worthwhile. In order to resolve some of the more serious problems it may be necessary to carry out additional measurements or structure refinements. However, the purpose of your study may justify the reported deviations and the more serious of these should normally be commented upon in the discussion or experimental section of a paper or in the "special_details" fields of the CIF. checkCIF was carefully designed to identify outliers and unusual parameters, but every test has its limitations and alerts that are not important in a particular case may appear. Conversely, the absence of alerts does not guarantee there are no aspects of the results needing attention. It is up to the individual to critically assess their own results and, if necessary, seek expert advice.

Publication of your CIF in IUCr journals

A basic structural check has been run on your CIF. These basic checks will be run on all CIFs submitted for publication in IUCr journals (*Acta Crystallographica, Journal of Applied Crystallography, Journal of Synchrotron Radiation*); however, if you intend to submit to *Acta Crystallographica Section C* or *E* or *IUCrData*, you should make sure that full publication checks are run on the final version of your CIF prior to submission.

Publication of your CIF in other journals

Please refer to the *Notes for Authors* of the relevant journal for any special instructions relating to CIF submission.

PLATON version of 18/09/2020; check.def file version of 20/08/2020

Datablock 2j - ellipsoid plot

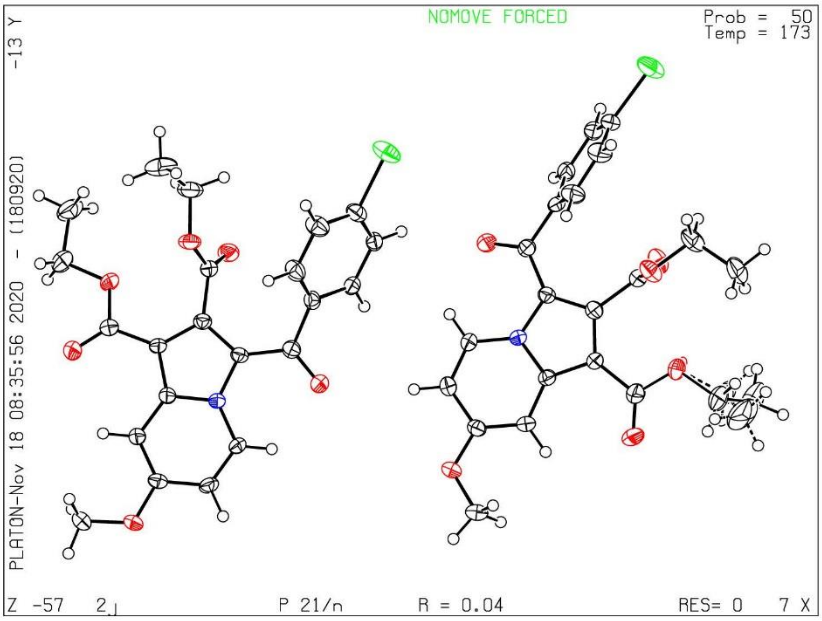

Supplement: Supplementary file 1 [file molecules-26-03550-s001.zip › molecules-1217846-supplementary.pdf]
